# Multiple Candidate Effectors from the Oomycete Pathogen *Hyaloperonospora arabidopsidis* Suppress Host Plant Immunity

**DOI:** 10.1371/journal.ppat.1002348

**Published:** 2011-11-03

**Authors:** Georgina Fabro, Jens Steinbrenner, Mary Coates, Naveed Ishaque, Laura Baxter, David J. Studholme, Evelyn Körner, Rebecca L. Allen, Sophie J. M. Piquerez, Alejandra Rougon-Cardoso, David Greenshields, Rita Lei, Jorge L. Badel, Marie-Cecile Caillaud, Kee-Hoon Sohn, Guido Van den Ackerveken, Jane E. Parker, Jim Beynon, Jonathan D. G. Jones

**Affiliations:** 1 The Sainsbury Laboratory, John Innes Centre, Norwich, United Kingdom; 2 School of Life Sciences, Warwick University, Wellesbourne, United Kingdom; 3 Warwick Systems Biology, Warwick University, Coventry, United Kingdom; 4 Biosciences, College of Life and Environmental Sciences, University of Exeter, Exeter, United Kingdom; 5 John Innes Centre, Norwich, United Kingdom; 6 Laboratorio Nacional de Genomica para la Biodiversidad, CINVESTAV Irapuato, Mexico; 7 National Research Council Canada, Plant Biotechnology Institute, Saskatoon, Canada; 8 Plant-Microbe interactions, Department of Biology, Utrecht University, Utrecht, and Center for Biosystems Genomics, Wageningen, The Netherlands; 9 Department of Plant-Microbe Interactions, Max-Planck Institute for Plant Breeding Research, Cologne, Germany; Massachusetts General Hospital, Harvard Medical School, United States of America

## Abstract

Oomycete pathogens cause diverse plant diseases. To successfully colonize their hosts, they deliver a suite of effector proteins that can attenuate plant defenses. In the oomycete downy mildews, effectors carry a signal peptide and an RxLR motif. *Hyaloperonospora arabidopsidis* (*Hpa*) causes downy mildew on the model plant *Arabidopsis thaliana (Arabidopsis)*. We investigated if candidate effectors predicted in the genome sequence of *Hpa* isolate *Emoy2* (HaRxLs) were able to manipulate host defenses in different *Arabidopsis* accessions. We developed a rapid and sensitive screening method to test HaRxLs by delivering them via the bacterial type-three secretion system (TTSS) of *Pseudomonas syringae* pv *tomato* DC3000-LUX (*Pst-LUX*) and assessing changes in *Pst-LUX* growth *in planta* on 12 Arabidopsis accessions. The majority (∼70%) of the 64 candidates tested positively contributed to *Pst-LUX* growth on more than one accession indicating that *Hpa* virulence likely involves multiple effectors with weak accession-specific effects. Further screening with a *Pst* mutant (ΔCEL) showed that HaRxLs that allow enhanced *Pst-LUX* growth usually suppress callose deposition, a hallmark of pathogen-associated molecular pattern (PAMP)-triggered immunity (PTI). We found that HaRxLs are rarely strong avirulence determinants. Although some decreased *Pst-LUX* growth in particular accessions, none activated macroscopic cell death. Fewer HaRxLs conferred enhanced *Pst* growth on turnip, a non-host for *Hpa*, while several reduced it, consistent with the idea that turnip's non-host resistance against *Hpa* could involve a combination of recognized HaRxLs and ineffective HaRxLs. We verified our results by constitutively expressing in *Arabidopsis* a sub-set of HaRxLs. Several transgenic lines showed increased susceptibility to *Hpa* and attenuation of *Arabidopsis* PTI responses, confirming the HaRxLs' role in *Hpa* virulence. This study shows TTSS screening system provides a useful tool to test whether candidate effectors from eukaryotic pathogens can suppress/trigger plant defense mechanisms and to rank their effectiveness prior to subsequent mechanistic investigation.

## Author Summary

*Hyaloperonospora arabidopsidis* (*Hpa*) is an obligate biotroph whose population coevolves with its host, *Arabidopsis thaliana*. The *Hpa* isolate *Emoy2* genome has been sequenced, allowing the discovery of dozens of secreted candidate effectors. We set out to assign functions to these candidate effectors, investigating if they suppress host defenses. We analyzed a sub-set of *Hpa* candidate effectors (HaRxLs) that carry the RxLR motif, using a bacterial system for *in planta* delivery. To our surprise, we found that most of the HaRxLs enhanced plant susceptibility on at least some accessions, while few decreased it. These phenotypes were mostly confirmed on *Arabidopsis* transgenic lines stably expressing HaRxLs that became more susceptible to compatible *Hpa* isolates. Furthermore, effectors that conferred enhanced virulence generally suppressed callose deposition, a hallmark of plant defense. This indicates that the “effectorome” of *Hpa* comprises multiple distinct effectors that can attenuate *Arabidopsis* immunity. We found that many HaRxLs did not confer enhanced virulence on all host accessions, and also that only ∼50% of the effectors that conferred enhanced *Pst* growth on *Arabidopsis*, were able to do so on turnip, a non-host for *Hpa*. Our data reveal interesting HaRxLs for detailed mechanistic investigation in future experiments.

## Introduction

Plants face constant attacks by a wide array of microorganisms including bacteria, fungi and oomycetes. Obligate biotrophic pathogens are particularly interesting because they can effectively evade or suppress host recognition, thus thwarting host defenses and enabling pathogen growth and reproduction [1].

In natural environments, plant disease is rare because plants activate a multilayered defense to most potential pathogens [2]. Relatively conserved molecules, called pathogen (or microbe)-associated molecular patterns (PAMPs), are recognized by the plants via pattern recognition receptor proteins (PRRs) [3], [4]. This interaction results in pattern-triggered immunity (PTI). Successful pathogens target effector proteins to the host cell cytoplasm to suppress PTI [5]. To counteract this, plants have evolved a second line of defense comprising resistance (R) proteins that recognize particular effectors either directly or through their activities on plant targets. This recognition leads to effector-triggered immunity (ETI) [2], [5].

It has been proposed that the “effector repertoire” of a given pathogen specifies its ability to infect a given host genotype [6], [7], [8]. Recent publications report many effector candidates predicted in the genomes of filamentous obligate biotrophs [9], [10], [11]. Comparison of effector sets of phylogenetically related species of obligate biotrophs that grow on different hosts reveals little overlap, suggesting host species-specific adaptation [10]. However, there are few studies about the functionality of obligate biotroph effectors on their hosts [12].

The downy mildew *Hyaloperonospora arabidopsidis* (*Hpa*) is an obligate biotroph that can only grow on *Arabidopsis thaliana* [13]. The *Hpa-Arabidopsis* pathosystem has been used to study host/parasite co-evolution and allowed the identification of cognate avirulent (AVRs) and resistance (R) proteins involved in specific *Arabidopsis*/*Hpa* interactions [14], [15]. The sequencing of the *Hpa* isolate *Emoy2* genome revealed its potential to encode at least 134 candidate effectors (HaRxLs) [9]. We report here assessments of the contribution of many of these HaRxLs to *Arabidopsis* immunity suppression.

Filamentous pathogens likely secrete their effectors from intercellular hyphae or haustoria [16]. Several studies have defined apoplastic and cytoplasmic effectors, based on their target sites in the host [17], [18], [19]. Cytoplasmic effector proteins have been inferred from either their localization inside the host cell (e.g. *Uromyces fabae* RTP1 protein) [20] or their recognition by host cytoplasmic R proteins; examples include *Melampsora lini* AVRs (AvrL567, AvrM, AvrP123, AvrP4), *Leptosphaeria maculans* (AvrLm1) and *Blumeria graminis* f.sp. *hordei* (AVRa_10_, AVRk_1_) [21], [22], [23]. In oomycetes, the cloning of four *AVR* genes, *Avr1b-1* (*Phytophthora sojae*), *Avr3a* (*Phytophthora infestans*, *P.i.*), *ATR1* and *ATR13* (*Hpa*) ([24], [25], [26], [27]) revealed a common N-terminal organization with signal peptides, enabling secretion from the pathogen, followed by a region that includes the amino acid motifs RxLR (for arginine (Arg), any amino acid, leucine (Leu), Arg) and EER (for glutamine (Glu, Glu, Arg) [28]. Functional analysis of Avr3a demonstrated that it accumulates in and is secreted from *P.i.* haustoria before its translocation into the host cell and its RxLR and EER motifs are required for delivery [29]. Avr1b requires its RxLR and EER motifs for uptake independently of the presence of the pathogen [30]. Binding of the RxLR EER and RxLR-like motifs of several fungal and oomycete proteins to phosphatidyl-inositol 3-phosphate (PI-3-P) has been proposed to mediate their entry into host cells [31]. In summary, the oomycete and fungal RxLR-like motifs, and the recently described LXLFLAK motif in Crinkler proteins [32] are conserved sequences involved in effector translocation into the host [33], [34]. For *Hpa*, no apoplastic effectors have been reported and the few effector candidates of *Hpa* that have LXLFLAK motifs, also carry overlapping RxLR motifs. For that reason we focused our “effectoromics” studies on predicted HaRxL-type effector candidates.

Unlike *Phytophthora* spp., *Hpa* is not transformable [35], [36]. Previous reports indicate that the bacterial type-three secretion system (TTSS) can be used to study how non-bacterial effectors can manipulate host cell functions [37], [38]. The phytopathogenic bacterium *Pseudomonas syringae* possesses a TTSS that translocates effectors to the host cell cytoplasm [39] via signals located on their N-termini [40]. *P. syringae* pv *tomato* DC3000 (*Pst* DC3000) grows on multiple *Arabidopsis* accessions [41]. Its growth *in planta* increases in PTI-compromised mutants like *fls2*, *cerk1*, *sdf2*, and *crt3* [42], [43], [44], [45], and decreases due to ETI when it delivers bacterial AVRs in plants carrying the cognate R proteins [46], [47], [48], [49]. The *Hpa* effectors ATR1 and ATR13 can be delivered from *P. syringae* using fusions to the N-terminus of the bacterial effectors AvrRps4 and AvrRpm1 [37], [50]. This technique has enabled the study of *Hpa* cytoplasmic effectors by monitoring growth *in planta* of *P. syringae* delivering different alleles of ATR1 and ATR13 into *Arabidopsis* accessions that carry (or not) the cognate R proteins RPP1 and RPP13. Although enhanced pathogen growth due to interference with host defence can be detected, it is likely that effectors whose prime role is to promote the elaboration of haustoria would be missed in this kind of assay.

By genomic and expression analysis of the *Hpa* isolate Emoy2 we defined 140 HaRxLs that carry a signal peptide and RxLR motif, and ranked them taking into account allelic diversity and expression level. Our aim was to survey a broad set of candidate HaRxLs to investigate if they might play a role in suppressing PTI and/or ETI. For this purpose the Effector Detector Vector (EDV) system [37], with a luciferase-expressing *Pst* DC3000 strain (*Pst*-LUX), was used for an initial assessment of whether 64 of these HaRxLs could enhance *Pst*-LUX growth on at least some *Arabidopsis* accessions. The majority of HaRxLs were found to increase host susceptibility on multiple accessions revealing a correlation with increased callose suppression. Interestingly, many HaRxLs were not effective on all accessions, implying that host effector targets might evolve to be refractory to effector action. However, although a few HaRxLs reduced bacterial growth on certain accessions, avirulence was rare. Selected HaRxLs were studied in more detail in transgenic plants, confirming their disease-promoting activities. On turnip, a non-host plant for *Hpa*, fewer HaRxLs enhanced *Pst*-LUX growth, and more reduced it, providing interesting clues into mechanisms that underpin non-host resistance. In addition to providing novel insights into how parasites impose host susceptibility, these data reveal several high priority HaRxLs for future mechanistic investigations.

## Results

### Identification of HaRxLs in the *Hpa* Emoy2 genome

**Figure 1:**
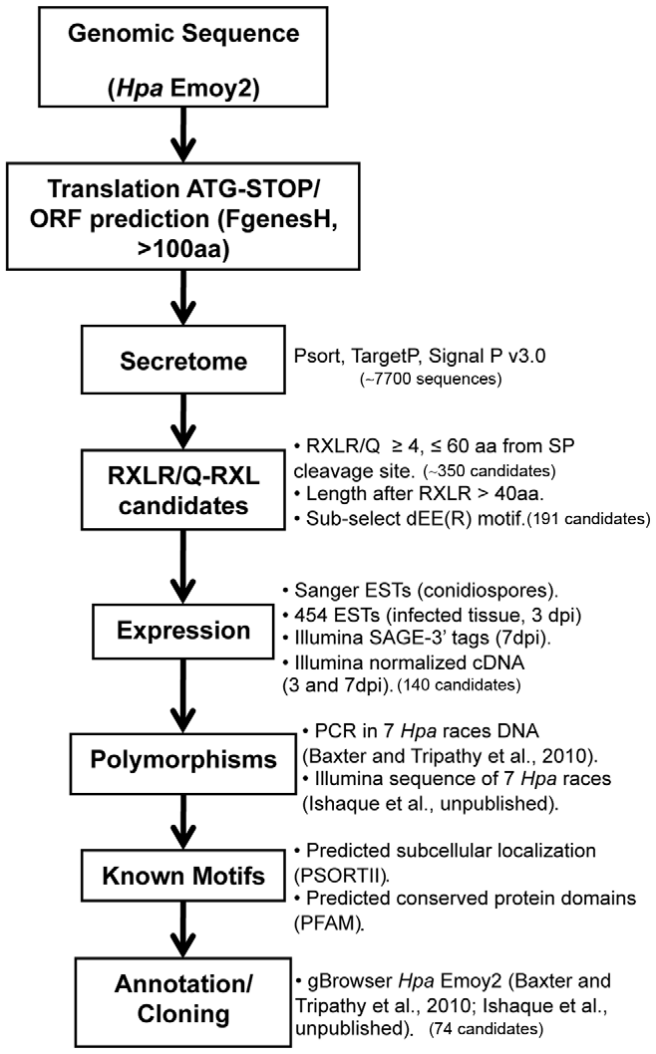
Bioinformatic pipeline used for the identification of *Hyaloperonospora arabidopsidis* (*Hpa*) HaRxLs. (*) The genome browser is maintained at the Sainsbury Laboratory (gbrowse2.tsl.ac.uk/cgi-bin/gb2/gbrowse/hpa_emoy2_publication).

To establish an inventory of the RxLR effector secretome of *Hpa*, we scanned the draft genome of Emoy2 (http://vmd.vbi.vt.edu) for all possible open reading frames (ORFs) encoding putative proteins longer than 100 amino acids. We then searched these sequences for the presence of signal peptides and from those we extracted gene models carrying RxLR-like motifs (RxLR/Q; RxL) with the sequence and positional constrains defined in Figure 1 (see Materials and Methods). Different sets of HaRxLs were identified depending on the version of the genome used (versions 3.0, 6.0 and 8.3.2). We merged different lists to define a set of 191 HaRxL genes that included the known effector genes ATR1 [26] and ATR13 [27]. Most of the encoded proteins were smaller than 300 amino acids. Approximately 37% had an acidic motif (EE/EER) after the RxLR and ∼6% had a predicted nuclear localization signal (data not shown). This collection included HaRxLs identified by others using similar search algorithms [9], [28].

We next tested which HaRxLs were expressed during the oomycete life cycle and whether these were correctly predicted. A set of Sanger ESTs from germinating *Hpa* spores ([9] and two different cDNA libraries from infected *Arabidopsis* plants at 3 and 7 days post inoculation (dpi) were used (see Materials and Methods). We verified expression of 140 of the 191 predicted HaRxLs. Ninety of them were expressed from the asexual spore stage, perhaps ensuring their early availability upon initiation of infection, and remained expressed *in planta* until 7 dpi. The remaining 51 HaRxLs were expressed either at 3 dpi, 7 dpi or both. Data in column M of Table S1 illustrate the expression pattern of the sub-sets of effectors tested in this work. Of those for which we could confirm expression, the majority (90%) of the HaRxLs was correctly predicted and none had introns (data not shown). Highly expressed and accurately predicted HaRxLs were prioritized for cloning.

We then looked for evidence of polymorphism in effector candidates between seven *Hpa* isolates (Cala2, Emco5, Emoy2, Hind2, Maks9, Noco2, Waco9). Single Nucleotide Polymorphisms (SNPs) were detected either on PCR products (Baxter et al., unpublished data) or partial assemblies of Illumina short reads (N. Ishaque, unpublished data). Our results indicated that 12% of the HaRxLs were not polymorphic, 56% had between 1 and 10 SNPs, and 31% showed more than 10 and up to 38 SNPs. We classified them as not polymorphic (0 SNPs), low (≥1 SNPs ≤5), medium (≥6 SNP_S_ ≤15) and high polymorphic (>16 SNPs) candidates (Column L, Table S1). For some HaRxLs it was difficult to distinguish heterozygosity from paralogous family members. In consequence, the real level of polymorphism might be underestimated. Recognized *Hpa* effectors like ATR1 and ATR13 show high levels of polymorphism [26], [27] while we hypothesize that non-recognized virulent effectors, adapted to interact with a specific host target, might have low sequence variability. Hence, candidates belonging to all four above described categories were used in this study.

### Construction and verification of an HaRxL library for functional screening using the EDV system

The Effector Detector Vector (EDV) delivers individual effector candidates to host plant cells using the TTSS of *Pseudomonas syringae* [37]. Seventy-four HaRxLs were cloned into pENTR/pEDV vectors (pEDV-HaRxLs) (Table S1). We obtained 71 fusion proteins (AvrRPS4N_1–136_–HA tag-HaRxL). Two candidate effectors could not be cloned correctly in pEDV, and another one was truncated and further used as a negative control (NC2, Table S1). Correct in-frame constructs were introduced by conjugation into *Pst* DC3000 and derivative strains, particularly one expressing the luciferase (luxCDABE) operon of *Photorhabdus luminescens* (*Pst*-LUX) [41] (see Materials and Methods for full details). No differences in bacterial growth (either in liquid or solid media) were observed in *Pst*-LUX clones carrying any of the 71 AvrRPS4N_1–136_–HA tag-HaRxL fusion proteins regarding the growth of *Pst*-LUX harbouring AvrRPS4N_1–136_–HA tag-GFP/AvrRPS4^AAAA^ (data not shown).

We performed *in vitro* secretion assays to check that the 71 fusion proteins obtained were made in bacteria and secreted to the medium in TTSS-inducing conditions. Secreted protein could be detected as illustrated in Figure S1A. Proteins of the expected size were produced by *Pseudomonas* for 64 of the pEDV5/6-HaRxLs cloned. No proteins, or protein bands of incorrect size, were observed for the remaining 7 HaRxLs, which were not used in further assays (Table S1, column H). Thus, our library comprised 64 Emoy2 pEDV-HaRxLs.

In *Pst* DC3000, the TTSS, encoded by the *hrp*-*hrc* (*h*ypersensitive *r*esponse [HR] and *p*athogenicity-*hr c*onserved) gene cluster, is required for elicitation of HR in non-host plants like tobacco and for full pathogenicity in host plants like tomato [51], [52]. To verify that the HaRxLs proteins did not alter *Pseudomonas* growth *in planta* by blocking the TTSS, we performed HR cell death tests in tobacco (*Nicotiana tabacum* cv. Petit Havana) and disease assays in tomato (*Solanum lycopersicum* cv. Moneymaker). Of the 64 pEDV-HaRxLs delivered by *Pst*-LUX in tobacco, only 1 attenuated HR in tobacco while four reduced disease symptoms in tomato. No candidate impaired both activities or completely abolished HR or disease (Table S2 columns C, D and representative examples in Figure S1 B, C). We infer from these results that none of the pEDV-HaRxLs constructs blocked *Pst*-LUX TTSS translocation of effectors.

The *Pst*-LUX strain was designed for screening multiple *Arabidopsis* mutants/accessions that vary in resistance to *Pst*DC3000 [41]. To evaluate the sensitivity of this system, we carefully validated the correlation between the level of bacterial bioluminescence and bacterial growth *in planta* using ATR1 and ATR13 (Figure S2). ATR1^Emoy2/Cala2^ and ATR13^Emoy2/Emco5^ conferred enhanced growth to *Pst*-LUX in the susceptible genotype Col-0, as did ATR13^Emoy2^ on Nd-0 plants. This phenotype could be detected as an increase in bioluminescence that correlated with higher numbers of bacteria (colony forming units (cfu)/cm^2^) (Figure S2). We were also able to detect decreased growth conferred to *Pst*-LUX by ATR13^Emco5^ or ATR1^Emoy2^ in Nd-0 plants (Figure S2).

### Several HaRxLs delivered via *Pst*-LUX increase bacterial growth on multiple *Arabidopsis* accessions

**Figure 2:**
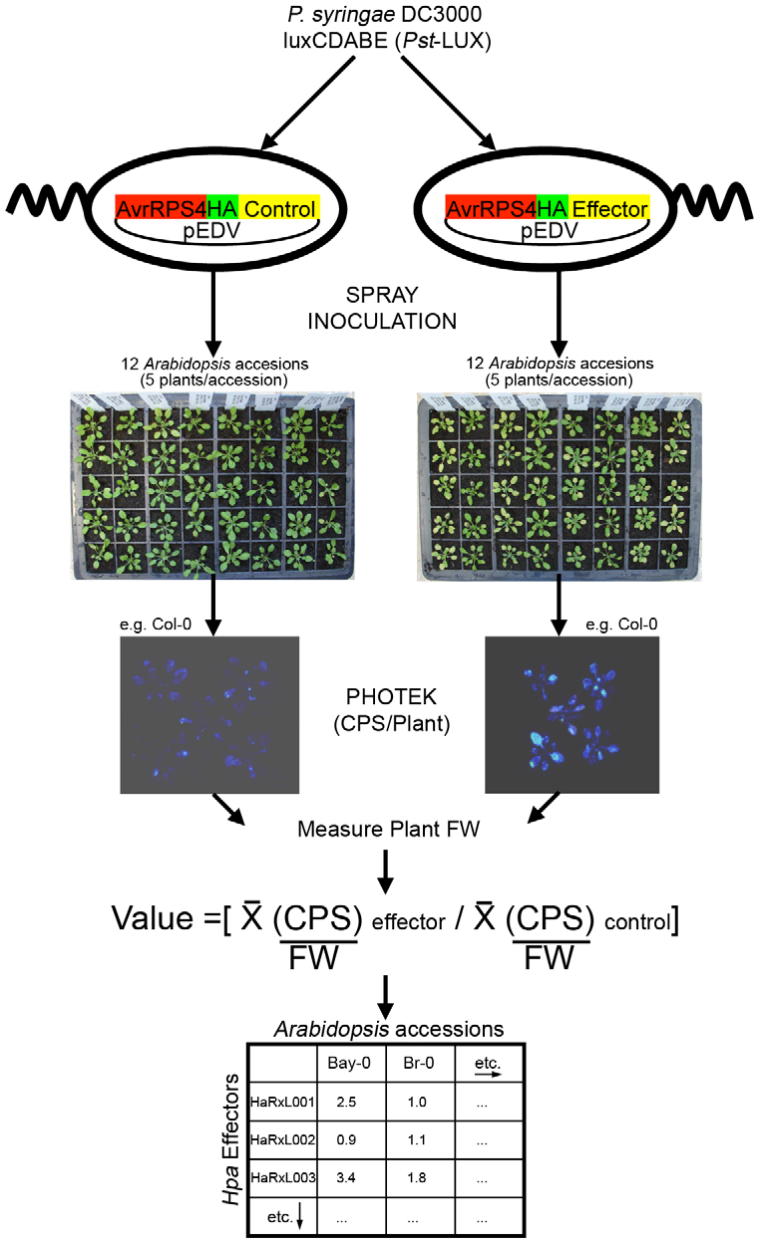
Functional screening method. *Hpa* effector candidates (HaRxLs) were delivered on 12 *Arabidopsis* accessions through the bacterial TTSS of the *Pst*-LUX strain. Levels of bacterial growth were measured quantifying bioluminescence (photon counts) emitted by the bacteria present on whole plants. The ratio of the average photon counts per second (CPS) per gram of fresh weight (FW) emitted by the bacteria delivering a given HaRxL versus the bacteria delivering control proteins was determined per accession. Experiments were repeated at least three times and statistical tests applied. Results and conclusions are shown in Table S2 and Figure 3.

Using spray inoculation, a protocol was developed to assay bacterial growth in a sub-set of the 96 accessions described by Nordborg et al., [53], selected to maximize variability (Bay-0, Br-0, Col-0, Ksk-1, Ler-0, Nd-0, Oy-0, Shakdara, Ts-1, Tsu-0, Wei-0, Ws-0) (Figure 2). Plants were spray-inoculated with *Pst*-LUX carrying EDV constructs that delivered either a control protein or an HaRxL via TTSS. At 3 dpi, the bioluminescence (photons/fresh weight) emitted by the bacteria was quantified as a measure of bacterial growth (Figure 2, see details in Materials and Methods). The growth of *Pst*-LUX *in planta* carrying each HaRxL was compared to a control (see below) and expressed as a ratio. This assay allowed us to establish whether a given HaRxL was able to enhance or decrease *Pst*-LUX growth, manifested as quantitative differences in bioluminescence, on multiple host accessions in parallel.

**Figure 3:**
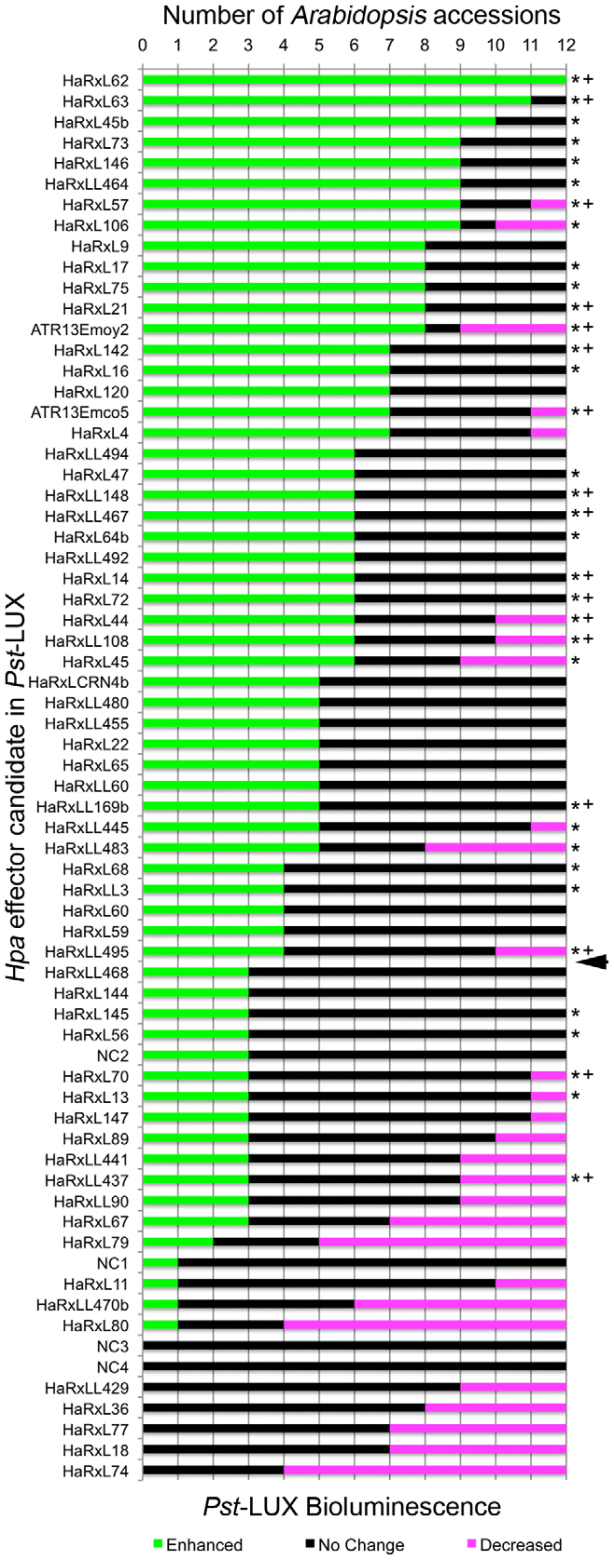
*Hpa* HaRxLs can promote or decrease *Pst*-LUX growth in different *Arabidopsis* accessions. The graph illustrates the outcome of the interaction between 12 *Arabidopsis* accessions (X axis) and *Pst*-LUX clones delivering 64 different *Hpa* effector candidates (HaRxLs, Y axis). Bars indicate the number of host accessions where the delivery of a given *Hpa* RxLR-like candidate effector by *Pst*-LUX conferred either enhanced (green bars), decreased (magenta bars) or no change (black bars) in bacterial growth, measured as bioluminescence, compared to the controls. The arrow indicates the threshold set up to consider that a given HaRxL truly enhances *Pst*-LUX bioluminescence. The asterisks indicate HaRxLs that suppress callose deposition in Col-0 when delivered via *Pst-*ΔCEL. High suppression levels are marked with (+). For details see Table S2, columns R,S and T. NC 1,2,3,4: negative internal controls.

Sixty-four pEDV-HaRxLs and 3 control proteins (EDV5:HA-AvrRPS4_1–136_, EDV6:HA-YFP, EDV6:HA-AvrRps4-^AAAA^) were delivered via *Pst-*LUX to 12 different *Arabidopsis* accessions. At three days post spray-inoculation, the photons/second/g fresh weight (CPS/Fw) were scored for five plants of each accession and averages, standard deviations and errors calculated. The ratio of increase or decrease in the CPS/Fw emitted by a *Pst*-LUX strain delivering a given pEDV-HaRxL, versus control (in the corresponding EDV5 or EDV6 backbone) was determined, as well as its statistical significance (one tailed T-test, unequal variances assumed) (Figure 2, Table S2). Experiments were repeated at least three times. Given the variability between experiments, the final outcome of each pEDV-HaRxL effect per accession was assessed across experiments and categorized according to the following criteria: i) a reproducible ratio higher or lower than one, showing the same trend on at least two experiments with a minimum statistical significance of p<0.05 on each of them, was considered as either “Enhanced” or “Decreased” growth and labeled with (+) or (−) respectively; ii) a non-reproducible ratio showing opposite statistically significant trends or the same trend but not statistically significant was considered as “No Change” and scored as ( = ) (see Table S2, columns R, S, T). A graphical synopsis of the screening outcome per effector across the 12 host accessions is presented in Figure 3, with the most effective pEDV-HaRxL (HaRxL62) at the top, conferring enhanced *Pst* growth on all 12 accessions.

To distinguish the effector-driven Enhanced/Decreased *Pst*-LUX growth patterns from the random phenotypes that can be obtained by delivering any given protein into the plant via the EDV system, we included four internal controls (negative controls, NCs). These constructs were truncated versions of HaRxLs (NC2 and NC3), non-secreted proteins with an RxLR-like motif (NC1) or a small bacterial protein with similarity to a xylosidase (NC4, genebank: AP12030.1). NC1 is part of a larger *Hpa* ORF encoding a putative transposase. NC2 is an early C-terminal truncated version of HaRxL143 (before the RxLR motif), while NC3 is a frame-shift version of HaRxL77 (Table S1). Functional ATR13^Emoy2^ and ATR13^Emco5^ alleles were also included. The pattern shown by these internal controls allowed us to establish threshold levels to assess whether a given HaRxL had a credible effect on *Pst*-LUX growth (Figure 3, Table S2). NC3 and NC4 did not impact *Pst*-LUX bioluminescence. We attributed the residual effect of NC1 and NC2 on *Pst*-LUX growth to the random variability of the system (Figure 3) and therefore we set the thresholds as follows: for an effector to be considered as an “enhancer” of *Pst*-LUX growth it had to show an increased significant change in bioluminescence in four or more accessions. Conversely, as the control ATR13^Emco5^ was recognized in only 1 accession out of the 12 tested, any effector that decreased the growth of *Pst*-LUX on one or more accessions was classified as capable of being recognized (Figure 3).

Our results indicate that 43 pEDV-HaRxLs enhanced *Pst*-LUX growth *in planta*, while 28 decreased *Pst-*LUX activity (Figure 3). The majority (72%) of the pEDV-HaRxLs that increased growth in ≥4 accessions did not decrease it in any accession (Green/black bars, Figure 3). This suggests most pEDV-HaRxLs can suppress plant defenses and avoid recognition by the host, although their effectiveness varies between accessions. We found only one pEDV-HaRxL capable of enhancing *Pst*-LUX growth in all accessions tested (HaRxL62); we infer that its plant target(s) might have little natural variation between accessions and that no R gene(s) recognize it in these accessions. In addition, pEDV-HaRxL9, 17, 21, 45b, 53, 73 and HaRxLL464 were able to increase *Pst*-LUX growth in 8 or more accessions and had no negative effect on the remaining ones. The pEDV-HaRxLs that decreased *Pst*-LUX growth did so mainly in ≤3 accessions (68%). This pattern of accession-specific *Pst*-LUX growth reduction was observed for both alleles of ATR13, and also for pEDV-HaRxL4, 44, 45, 57, 106, 108, HaRxLL445, 483 and 495. Only nine pEDV-HaRxLs conferred decreased bacterial growth in >3 and <8 accessions. Given the lack of accession-specificity of their phenotype we speculate these HaRxLs might affect the virulence activity of *Pst* effectors (Magenta areas, Figure 3).

### Verification via growth curves of HaRxL-induced changes in *Pst*-LUX growth highlights a subset of effective HaRxLs

We extended the concordance analysis developed with ATR1^Emoy2/Cala2^ and ATR13^Emoy2/Emco5^ (Figure S2) to 13 other pEDV-HaRXLs delivered from *Pst*-LUX by conducting growth curve assays using seven *Arabidopsis* accessions (Table S3). For this test we selected some HaRXLs representative of the different patterns we observed in Figure 3. Briefly, we tested HaRxL62 because it enhanced *Pst*-LUX luminescence in all accessions; HaRxL14, HaRxL21, HaRxLL60, HaRxLL464, and HaRxLL492 because they enhanced *Pst*-LUX luminescence in ≥6, but did not decreased it in any accession. HaRxL44, 45, 57 and 106 also increased bacterial luminescence in ≥6 accessions but decreased it in 1–3 accessions. HaRxL70 was selected among the group of “non-effective” effectors, and HaRxL79 because it reduced *Pst*-LUX bioluminescence in >3 accessions.

For 32 of 35 combinations (pEDV-HaRXL×accession) we confirmed the correlation between enhanced bioluminescence and increased bacterial growth. These data verified that *Pst*-LUX bioluminescence reveals the effect of HaRXLs on *Pst-*LUX growth. We also observed that some HaRXLs have a substantial positive effect on bacterial growth on multiple accessions, and can increase *Pst*-LUX growth ∼10-fold (Table S3 and Figure S3). In particular, we confirmed that HaRxL62 and HaRxL14 render multiple host accessions more susceptible to bacterial infection (Figure S3). Accession-specific effects were verified for HaRxLL464 and HaRxL21 while putative recognition events, leading to a decrease in bacterial growth, were verified for HaRxL44 in Ler-0, HaRxL57 in Ksk-1 (Figure S3, Table S3), and HaRxL106 in Col-0 (Table S3). No effect was observed for HaRxL70 in Col-0 while the decrease in bacterial growth caused by HaRxL79 was only observed when plants were spray inoculated (Table S3). These data reinforced the usefulness of the EDV *Pst*-LUX assay for selecting candidates for further work, and confirmed several candidates as a high priority for further investigation.

### Host genotypes and levels of HaRxL polymorphism are not correlated with effector-induced changes in *Pst*-LUX growth

To evaluate if host genotypes influenced the pattern of *Arabidopsis* responsiveness to the set of HaRxLs tested, the spectrum of effective HaRxLs per accession was analyzed. We found that an average of 42% of the pEDV-HaRxLs enhanced *Pst*-LUX growth on any given accession, while only ∼11% reduced *Pst-*LUX growth. Many combinations (46%) did not cause any change in *Pst-*LUX growth (Figure S4). Enhancement or decrease of susceptibility was not restricted to a particular set of accessions, and did not correlate with those accessions showing resistance or susceptibility to the infection by the *Hpa* isolate Emoy2 (Figure S4). The only deviations from this pattern were Nd-0, in which most of the pEDV-HaRXLs (73%) increased *Pst* growth and only ATR13^Emco5^ was able to decrease it, and Br-0 in which fewer pEDV-HaRXLs in total were effective (31% compared to the average of 42% for all other accessions) (Figure S4). These results are consistent with the idea that some effector targets are widely conserved while others vary between accessions.

The level of polymorphism of HaRxLs did not correlate with the capacity to enhance *Pst*-LUX growth. Among the 64 candidates tested, 11 were highly polymorphic, 21 had a medium level and 32 showed low polymorphism. HaRxLs categorized in these three groups showed ability to increase bacterial luminescence in an average of 6±2.54, 6±3.15 or 5±2.66 accessions, respectively. For example, HaRxLL464 and HaRxL57 showing low or no polymorphism, and the highly polymorphic HaRxL106 and HaRxL21 were all capable of increasing *Pst*-LUX growth in 8 or more host accessions.

### HaRxLs did not trigger hypersensitive recognition in any *Arabidopsis* accession after EDV delivery

Isolate Emoy2 is recognized by certain *Arabidopsis* accessions, indicating effector recognition by R protein(s). In order to identify avirulent HaRxLs in the library, we analysed in detail *Pst*-LUX growth assays in each of the 12 *Arabidopsis* accessions (Figure 3, Figure S4). Possible recognition of pEDV-HaRxL strains in our assays was indicated by the decrease in *Pst*-LUX growth, usually in an accession-specific manner (Figure 3, Table S2). Potentially novel ATR proteins may have been detected in interactions with accessions Col-0, Ler-0, Br-0 and Ksk-1 (Figure S4).

ETI is strongly correlated with HR-like cell death [54], [55] although HR is not always required for resistance [49], [56]. We tested possible recognitions using a weakly virulent *Pst* DC3000 ΔCEL (*Pst*-ΔCEL) strain and a modified *P fluorescens* carrying a functional TTSS (Pf0-1) [57] to deliver potentially recognized HaRxLs to the corresponding “resistant” accessions. We performed localized leaf infiltrations using high doses of bacteria and looked for macroscopic (leaf collapse) and microscopic (dead cells stained with trypan blue) indicators of HR-like cell death 24 h post infiltration.

Surprisingly, no HaRxLs besides the positive control (ATR13^Emco5^ in Nd-0) provoked clear signs of macroscopic HR. We then stained infiltrated leaves with trypan blue and examined for microscopic lesions. All micro-HR lesions were much smaller and weaker than those triggered by bacterial effectors like AvrRpm1 or AvrRpt2 (data not shown) or by the *Hpa* effector ATR13^Emco5^ in Nd-0 (Table S4). In 78 pEDV-HaRXL/accession combinations, we saw micro-HR-like cell death in only 7 interactions, comprising just 4 candidate effectors (HaRxL4, 18, 70 and 80; in bold in Table S4). Similar results were obtained with both Pf0-1 and *Pst*-ΔCEL strains, except for HaRxL106 where no HR was detected in Col-0 and Ksk-1 when delivered through Pf0-1 (Table S4). Nevertheless, the decrease in bacterial growth observed for *Pst*-LUX delivering each of these candidate effectors in the corresponding accessions was confirmed by reduction in disease symptoms and bacterial growth using *Pst*-ΔCEL (data not shown).

None of the mild recognition patterns matched with profiles expected for ATR4, ATR5 and the putative ATR(s) recognized in Ksk-1 and Br-0 (Figure S4). Interestingly, two HaRxLs (HaRxL18 and 70) were weakly recognized in Bay-0, an accession susceptible to isolate Emoy2. These results suggest that the decreases in *Pst* growth we see in some HaRxL/accession combinations are not due to strong R/AVR interactions. Also, weak recognition of some HaRxLs might not result in HR [49], [56].

### Importance of PTI suppression for *Hpa* infection

Many HaRxLs delivered *in planta* by *Pst*-LUX confer increased growth of an already virulent pathogen. Enhanced susceptibility to adapted pathogens is often a result of PTI suppression [42], [43], [44], [45]. PTI- responses, like callose deposition, likely limit the growth of *Hpa* during infection [58], [59], [60]. Also, ATR13^Emoy2/Emco5^ can complement HopM1-mediated suppression of callose deposition when delivered by *Pst*-ΔCEL [37]. Therefore, we investigated if PTI affects *Hpa* growth, and whether *Hpa* is able to actively suppress PTI. As no known PAMP has been identified for *Hpa*, we tested whether *Hpa* infection alters responses to known PAMPs.

**Figure 4:**
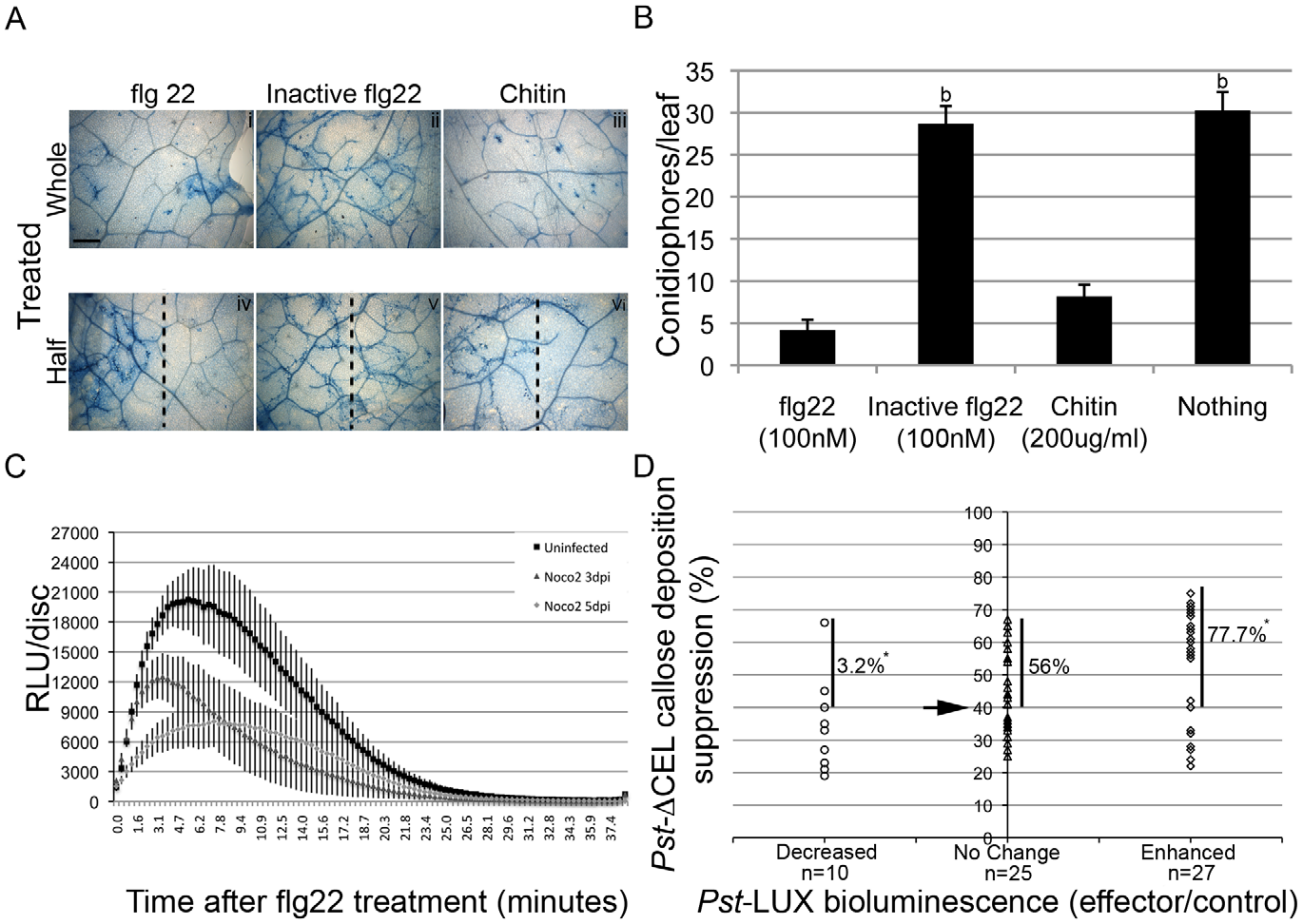
Suppression of PTI as a virulence tool for *Hpa*. (A) Pre-treatment of Col-0 leaves with flg22 or Chitin reduces *Hpa* isolate Noco2 hyphal colonization. Leaves of four-week-old Col-0 plants were pre-infiltrated with 100 nM flg22, inactive flg22 (from *A. tumefasciens*) or Chitin (200 µg/ml) 24 h before inoculation of *Hpa* Noco 2 (5×10^4^ sp/ml). Pictures show trypan blue stained leaves at 5 days post-*Hpa* spraying (dps). This experiment was repeated three times with similar results and also for Emoy2 on Oy-0 plants. Panels i, ii and iii: the whole area shown was pre infiltrated. Panels iv, v and vi: only the right side of the picture was infiltrated. Dotted vertical line indicates approximated infiltration boundaries. Bar is 500 µm. (B) Pre-Induced PTI responses reduce *Hpa* asexual reproduction. Leaves of three-week old Col-0 plants were infiltrated with the indicated solutions 24 h before infection with Noco2 (5×10^4^ sp/ml). Conidiophores per leaf were counted on trypan blue stained leaves excised at 5 dps. Bars represent the average ±2× SE of 40 leaves. This experiment was repeated three times with similar results. (b) p value <0.01, T-test. (C) *Hpa* infected tissues show reduced ROS response to flg22. Leaf discs from uninfected and infected Col-0 plants were treated with 100 nM flg22, and the level of ROS generated measured with a Luminometer. Values indicated are average of Relative Luminescence Units (RLUs) ± SE of 24 leaf discs. (D) HaRxLs delivered by *Pst-*ΔCEL in Col-0 plants suppress callose deposition. Effector's impact on the level of *Pst-*ΔCEL-triggered callose deposition is presented in the Y-axis. The average reduction (in percentage) of callose deposits observed when a given candidate effector was delivered, compared to the number of callose deposits observed when control proteins were delivered by *Pst-*ΔCEL, is represented by the shapes in the body of the graph. HaRxLs were also categorized according to their phenotype on *Pst-*LUX bioluminescence in Col-0 (X-axis). The arrow indicates the threshold set up to consider callose deposition as significantly suppressed. The numbers in the body of the graph correspond to the percentage of HaRxLs able to suppress callose deposition among each bioluminescence category. (*) Indicates p<0.05 of Z-test versus random distribution expected for the number (n) of HaRxLs on each group.

To test if PTI can attenuate *Hpa* growth during a compatible interaction, we pre-treated young Col-0 plants with flg22 (100 nM), an inactive flg22 (from *Agrobacterium tumefaciens*), or chitin (200 µg/ml) 24 h before the plants were sprayed with spores of *Hpa* isolate Noco2. Reduced hyphal growth was observed in the areas where the PAMPs were applied, as assessed by trypan blue staining of the pathogen in the leaves (Figure 4 A). We also noticed a decrease in the rate of *Hpa* sporulation (Figure 4 B). These phenotypes were not observed with inactive flg22 (Figure 4 A, B), or when the treatment was applied on plants mutant for these PAMP receptors (fls2-1 and cerk1-1, data not shown). These data suggest that the phenomenon is specific for PTI. We also noticed that the “protection” that flg22 and chitin conferred to the plants was higher near the infiltrated site, was dose-dependent, and diminished with the time of pre-infiltration relative to *Hpa* infection (24 hs>48 hs>72 hs) (Figure 4 A lower panel and data not shown), consistent with the transient nature of the local PTI response [61]. We did not observe extensive local micro-HR in flg22-treated leaves [62]. The “local” effect of flg22 and chitin in restricting *Hpa* hyphal growth (Figure 4 A) might indicate that either we did not induce systemic acquired resistance (SAR) [63], or we applied *Hpa* before SAR was established.

One of the earliest PTI responses is the generation of reactive oxygen species (ROS burst) [64]. To determine if *Hpa* can suppress ROS, we measured flg22-induced ROS in infected leaf tissues. We observed a highly reproducible reduction by ∼50% in ROS accumulation induced by flg22 if leaves were pre-infected with *Hpa* isolate Noco2 (Figure 4C). Oy-0 plants infected with Emoy2 showed the same pattern (data not shown). Thus, *Hpa* infection can dampen PTI responses.

### Most HaRxLs that increase bacterial growth in Col-0 can suppress *Pst*-ΔCEL-induced callose deposition

PTI results in callose deposition in the cell wall [65] and microbial effectors that impair PTI also suppress callose deposition [66], [67], [68], [69]. *Pst*-ΔCEL is unable to suppress callose deposition due to lack of HopM1 and AvrE [70]. We introduced pEDV-HaRxL constructs into *Pst*-ΔCEL and evaluated if they could restore callose suppression when infiltrated in Col-0.

Sixty-two pEDV-HaRxLs and 2 control proteins were delivered through this system. Due to variability between leaf reactions in the same plant and among experiments, we established a threshold to define significant reductions in callose deposition (see Materials and Methods). Taking into account the maximum levels of random callose suppression observed after delivery of the negative controls (NC1 and NC2), we set up the threshold to 40% of callose suppression because negative controls could reduce up to 29% or 34% of the callose dots found when *Pst*-ΔCEL delivered the additional controls (EDV5:HA-AvrRPS4_1–136_, EDV6:HA-YFP or EDV6:HA-AvrRps4^AAAA^). Using this stringent criterion we found that 35 HaRxLs were able to suppress callose deposition by more than 40%, while 27 HaRxLs did not. Those effectors complementing the phenotype of *Pst*-ΔCEL are indicated with asterisks (*) in Figure 3.

We noticed that most of the HaRxLs able to complement *Pst*-ΔCEL were also able to enhance *Pst*-LUX growth in four or more host accessions (Figure 3). To establish the degree of correlation between both phenotypes, the extent of callose suppression was compared with the changes in *Pst*-LUX growth produced by each effector in the accession Col-0 (Figure 4 D). For this, we classified the effector's conferred phenotype in the host as follows: enhanced susceptibility = 27 HaRxLs (right side of the graph), decreased susceptibilty = 10 HaRxLs (left side of the graph), no change = 25 HaRxLs (axes intersection). When we plotted the percentage of callose suppression of each of the effectors in these groups, we found 77.7% of HaRxLs candidate effectors with a positive effect on *Pst*-LUX growth in Col-0 were able to suppress callose deposition, while only 3.2% of those decreasing *Pst*-LUX growth could reduce callose levels (Figure 4 D). These percentages deviate significantly from those expected for a random distribution (Z-test p<0.05). The HaRxLs located at the top right side of Figure 4 D were those that strongly suppressed callose deposition, and are indicated with plus (+) signs in Figure 3. The HaRxLs with no effect on *Pst*-LUX growth showed no clear trend in ability to suppress callose deposition.

We conclude that most HaRxLs that enhance *Pst* growth also suppress PTI, increasing host-susceptibility. Based on the data presented in Figure 3, Tables S2 and S3, Figure S3 and Figure 4D, we prioritized HaRxL14, 21, 44, 45/45b, 57, 62, 106, HaRxLL60, 464, 492 and 495 for further detailed studies. HaRxL14, 21, 44, 57 and 62 were chosen because they increased *Pst*-LUX growth in more than 6 accessions and strongly suppressed callose deposition in Col-0 (>60%) (Figure 3 and top right side on Figure 4D). HaRxLL464 and HaRxL45/45b strongly enhanced *Pst*-LUX growth in 9 or more accessions, but showed a mild reduction in callose deposits (Figure 3 and Table S2). HaRxL106 conferred enhanced susceptibility to *Pst*-LUX in several ecotypes, except Col-0, where it nevertheless reduced callose deposits (Figure 3). HaRxLL60 and HaRxLL492 conferred enhanced growth of *Pst*-LUX in 5–6 accessions, but were unable to complement the *Pst*-ΔCEL phenotype.

### *Arabidopsis* transgenic lines constitutively expressing HaRxLs show PTI suppression and enhanced susceptibility

To investigate if the phenotypes observed with the *Pst*-EDV-delivery screenings were also conferred by stably expressing the corresponding HaRxLs directly in the host plant, transgenic *Arabidopsis* plants were generated initially in the Col-0 background. The following HaRxLs were expressed from the constitutive (CaMV 35S) promoter: HaRxL14, 21, 44, 45/45b, 57, 62, 106, HaRxLL60, 464, 492 and 495. Of these, for three candidates (HaRxL62 and HaRxL45/45b) either we did not obtain transgenic lines or the ones generated showed segregation of pleiotropic effects, and in consequence are not described here. Some plants (lines 35S-HaRxLL464 and 35S-HaRxL44) showed a 20–30% increase in fresh weight and others (line 35S-HaRxLL60) a 30–40% decrease in fresh weight. In some cases (35S-HaRxL106 and 35S-HaRxLL60) the shape of the leaves changed, becoming either elongated and darker, or serrated and smaller, respectively (data not shown). The level of expression of the transgene in each line was verified by semi-quantitative RT-PCR (Figure S5).

**Figure 5:**
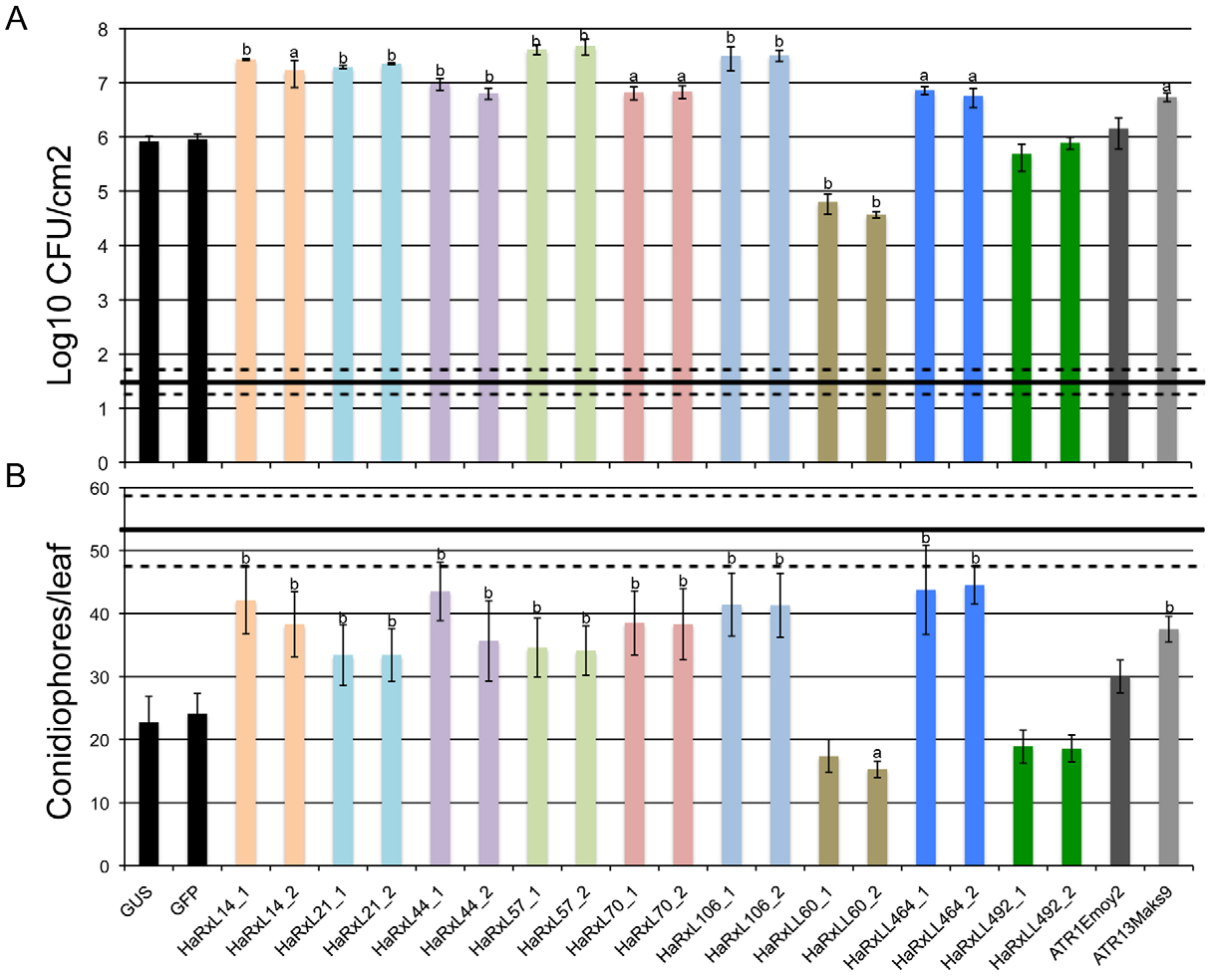
*Arabidopsis* Col-0 plants expressing constitutively HaRxLs support enhanced growth of *P. syringae* ΔavrPto/ΔavrPtoB and *Hpa* isolate Noco2. (A) Four leaves of three five-week-old plants of two independent transgenic lines per HaRxL were infiltrated with *Pst-*ΔavrPto/ΔavrPtoB at OD_600_ = 0.0005. Bacterial growth was determined at 3 dpi by traditional growth curve assays. Bacterial populations immediately after inoculation (3 h; 0 dpi) were averaged among plants and are represented by the solid black horizontal line, with 2× SE represented by the dashed horizontal lines. (a) T-test p value<0.05, (b) T-test p value<0.01.This experiment was repeated two times with similar results. (B) Two-week-old seedlings were spray inoculated with a suspension of 1×10^4^ conidiospores per ml of *Hpa* isolate Noco2. At 6 dps, whole seedlings were cut and stained with Trypan blue. The number of conidiophores per leaf was counted in 4 leaves per seedling. Ten seedlings were analyzed per transgenic line per HaRxL. The horizontal black and dashed lines represent the average ±2× SE of the number of conidiophores per leaf found in the hyper-susceptible mutant Col-0 *eds1-2*. (a) T-test p value<0.01, (b) T-test p value<0.05. This experiment was repeated three times with similar results.

Using three independent lines for the remaining nine candidate effectors, that did not showed perturbed growth, we assessed whether *in planta* over-expression altered pathogen development, PTI or ETI. We characterized the responses of these transgenic lines to both bacterial (*Pst*-LUX, *Pst* ΔavrPto/ΔavrPtoB) and oomycete pathogens (*Hpa* isolates Noco2 and Emoy2) (Figure 5 and Figure S6). Transgenic lines expressing different HaRXLs showed increased susceptibility to *Pst*-LUX when spray-inoculated (8 lines, Figure S6), or to *Pst* ΔavrPto/ΔavrPtoB when infiltrated (7 lines, Figure 5 A). Also, seven lines showed enhanced susceptibility to *Hpa* isolate Noco2 (Figure 5 B). These phenotypes were observed in at least two out of the three transgenic lines recovered for each effector.

**Figure 6:**
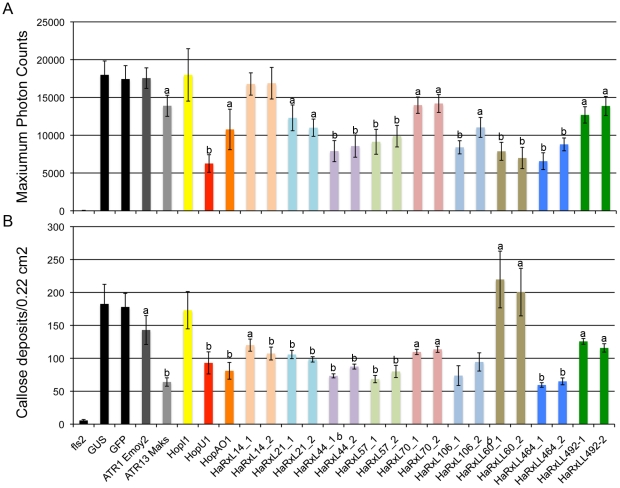
*Arabidopsis* plants expressing constitutively HaRxLs accumulate less ROS and/or callose in response to flg22. (A) Leaf discs from four-week-old transgenic plants expressing the indicated HaRxL were sampled and floated in water 14 to 16 h prior to flg22 treatment. Photon emission was measured every 100 milliseconds for 40 minutes. Lines and error bars represent the mean of maximum values of photon counts ±2× SE of 24 independent leaf discs. This experiment was repeated four times with similar results. (B) Leaves of four-week-old transgenic lines were hand inoculated with 100 nM of flg22. Twenty-four hours post-inoculation, leaf discs were sampled and stained with aniline blue for visualization of callose dots. The bars represent mean ±2× SE of callose dots per image photographed (field of 0.22 square centimeters). Callose dots were quantified with ImageJ. Twenty leaf discs were analyzed per transgenic line. This experiment was repeated three times with similar results.

**Figure 7:**
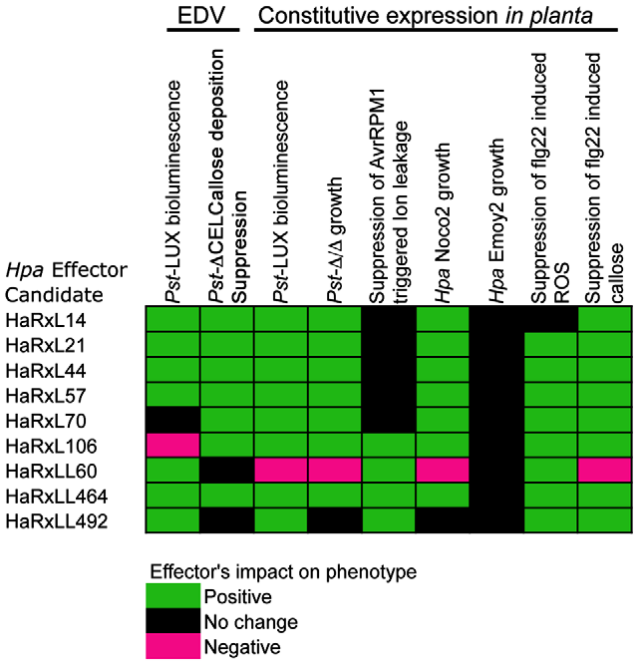
Summary of phenotypes observed upon expression of HaRxL effectors in *Arabidopsis*. Graphical comparison of the results obtained using the transient EDV assays and stable constitutive expression for nine different HaRxLs. The phenotypes analyzed include bioluminescence of *Pst*-LUX, suppression of callose deposition triggered by *Pst-* *Δ*CEL, growth of *Pst-* *Δ*avrPto/*Δ*avrPtoB, suppression of ion leakage triggered by delivering AvrRPM1 via Pf0-1, growth (conidiation) of *Hpa* compatible (Noco2) and incompatible (Emoy2) isolates, and suppression of the levels of ROS and callose deposition triggered by flg22 treatments.

We investigated if the transgenic lines were compromised in ROS burst and callose deposition in response to flg22 (Figure 6). Eight 35S-HaRxLs were able to reduce flg22-triggered ROS accumulation by 22 to 65% compared to controls (Figure 6 A). Also, callose deposition was diminished by an average of 40% compared to controls (Figure 6 B). The ROS and callose suppression in transgenic lines expressing HaRxL 14, 21, 44, 57, 106, HaRXLL 464 was comparable to that observed in plants that express the bacterial effectors HopU1 and HopAO1 (Figure 6 A, B). In summary, six different *Hpa* HaRxLs, when stably-expressed *in planta*, displayed a positive correlation between increased susceptibility to *Ps*t and/or *Hpa* and reduced levels of ROS and callose deposition elicited by flg22 (Figure 7).

To establish if any of the nine HaRxLs could also compromise ETI, we tested transgenic lines for altered resistance to *Hpa* Emoy2 which is recognized in Col-0 [71]. Two-week-old seedlings were sprayed with Emoy2 conidiospores and trypan blue-stained at 5 dpi. While some restricted hyphal growth was detected, we did not observe asexual or sexual reproduction -in true leaves- in any line (summarized in Figure 7 and data not shown). We then studied the ETI response to AvrRpm1 from *P. syringae* pv. *maculicola* [72]. AvrRpm1 was delivered via Pf0-1 in leaves of 4-week-old plants and a macroscopic HR recorded. The onset of HR was delayed but not completely suppressed in four different lines (data not shown). We therefore performed a more sensitive assay by quantifying the levels of ion leakage upon AvrRpm1 detection (Figure S7). Notably, the same four sets of transgenic lines (35S-HaRxL106, 35S-HaRxLL492, 35S-HaRxLL464 and 35S-HaRxLL60), slightly but significantly reduced the levels of ion leakage caused by recognition of AvrRpm1 compared to the control lines (Figure S7 and Figure 7).

Our results suggest that the majority (six out of nine) of *Hpa* Emoy2 HaRxLs constitutively expressed *in planta* gave phenotypes (enhanced susceptibility to *Pst*-LUX and suppression of PAMP-triggered callose deposition) consistent with the results obtained from the *Pst*-LUX EDV-screening in the Col-0 accession (Figure 7). Two transgenic lines (35S-HaRxL106 and 35S-HaRxLL60) showed phenotypes that contrasted with those observed in the EDV screen on Col-0, and for another line (35S-HaRxL70) no effect was detected with the EDV system in Col-0, while enhanced *Pst*-LUX growth was observed in the over-expressing plants (Figure 7). When HaRxL106 is delivered by EDV from *Pst* DC3000 strains, it appears to be recognized by Col-0 and Ksk-1 accessions, but this HR-like cell death is not evident when it is delivered by Pf0-1 (Table S4). Expression of 35S-HaRxLL60 caused the plants to be smaller, accumulate callose constitutively and become resistant to the pathogens tested (Figure 5, Figure 6B, Figure S6). Nevertheless, 35S-HaRxLL60 reduced flg22 ROS production (Figure 6A). The set of six lines with enhanced susceptibility to *Pst* and/or *Hpa* also displayed a reduced ROS burst and callose deposition after PAMP treatment (Figure 7). Overall, our analysis points to suppression of PTI-related responses as a predominant mode of action of *Hpa* candidate effectors *in planta*.

### Emoy2 HaRxL candidate effectors are mostly ineffective or reduce pathogen growth in the *Hpa* non-host *Brassica rapa*

Most plants are resistant to most pathogens, and this so called “non-host resistance” (NHR) could be caused by either ineffectiveness of effectors, resulting in failure to suppress PTI, or recognition of effectors, resulting in resistance via ETI [8]. To test these hypotheses we delivered HaRxLs via *Pst*-LUX in the non-host *Brassica rapa* cv Just Right (turnip).

*Pst*-LUX is virulent in turnip and causes disease symptoms at 3 dpi when inoculated at low dose. We tested our collection of HaRxL-carrying *Pst*-LUX strains in turnip and monitored symptoms and growth. After three rounds of screening we found that 20 effectors can alter *Pst*-LUX growth in turnip (13 increase, 7 decrease), while the remaining 44 did not cause any changes (Figure S8 and Table S2, column U). We compared these data with the number of “effective” HaRxLs in one given *Arabidopsis* accession (39 in Col-0) and the average for the 12 accessions we tested (35) (green plus magenta bars vs. black bars in Figure S4). In contrast, only thirteen HaRxLs increased *Pst*-LUX growth in turnip, while in *Arabidopsis* accessions an average of 27, and minimum of 18 (in Br-0) increased *Pst*-LUX growth (see Figure S4 and S8). Those candidates enhancing *Pst*-LUX growth in turnip also did so in >3 *Arabidopsis* accessions, implying that their effect on plant immunity is not species-specific and some plant targets might be conserved. Conversely, while similar numbers of HaRxLs decrease *Pst*-LUX growth in *Arabidopsis* (8 in average) and turnip (7), we noticed three HaRxLs (HaRxL17, HaRxL47 and HaRxL63) that reduced *Pst*-LUX growth and disease symptoms in turnip that did not show this phenotype in the 12 *Arabidopsis* accessions. To assess if these HaRxLs might be specifically recognized in turnip and contributing to NHR against *Hpa*, we used higher dose *inocula* to deliver them using *Pst*-ΔCEL. We did not observe HR-like cell death, but we confirmed the reductions in growth and disease symptoms (data not shown). It remains possible that these HaRxLs might be triggering weak ETI in turnip that does not involve HR-like cell death but still contributes to NHR (see Discussion).

## Discussion

Genome sequences of plant pathogens have enabled searches for effectors that might manipulate host cells ([9], [28], [73], [74], [75], this work). Verified effectors provide molecular probes to investigate plant defense mechanisms and better understand pathogen adaptation to hosts. In *Hpa*, ∼134–140 HaRxLs have been identified [9], [28], this work] and the challenge is to identify those that are functional and then investigate their biological mechanisms.

For effector discovery, we combined the use of a heterologous system for *Hpa* candidate effector delivery from *Pst* DC3000, with a rapid and sensitive assay for bacterial growth *in planta*, and for suppression of callose synthesis. To independently assess the efficacy of the most promising candidates, we also tested the consequences of constitutively expressing selected HaRxLs *in planta*.

### EDV vectors and *Pst*-LUX strains provide a sensitive assay for effects of HaRxLs on plant immunity

We found that of 64 expressed HaRxLs that could be secreted by the EDV delivery system, 43 (∼67%) could increase the growth of *Pst*-LUX in several (>4) host accessions. We were surprised that so many candidate effectors can enhance growth of an already virulent pathogen. The false positive rate is likely to be low, while the false negative rate could be high, because we set a stringent threshold to judge a putative effector “effective” compared to four internal controls. Despite the intrinsic variability of *Pst*-LUX spraying assays, the phenotypes were reproducible, even when the measurable differences were small (Table S2 and S3). We found that detection of LUX activity is more sensitive and less laborious than growth curve assays and noticed that 2- fold differences in LUX emission usually corresponded to differences between 0.3 and 0.6 log in growth. These might be considered small contributions to virulence, but are consistent with previously reported observations for bacterial effectors such as AvrRpm1 AvrRpt2, AvrPtoB, HopF2, HopAO1 and HopU1, where their individual contribution to *Pst* DC3000 growth is of the same order of magnitude (around 0.4 log) or only detectable using low virulence *Pst* mutants [47], [69], [76], [77], [78].

A strong advantage of the EDV approach is that one *Pst*-DC3000 strain can be tested on many different *Arabidopsis* accessions to reveal accession-specific differences in HaRxL efficacy. This would be extremely laborious by generating stable transgenic lines in multiple accessions. Furthermore, some HaRxLs confer severe pleiotropic defects when expressed directly *in planta*, hampering efforts to test whether such lines are immunocompromised.

### *Hpa* virulence likely involves multiple effectors with weak accession-specific effects

Since not all HaRxLs are effective in all accessions, it seems likely that each *Hpa* isolate expresses a repertoire of effectors, each of which may be functional on some but not all host genotypes. The level of infection in Col-0 by compatible *Hpa* isolates is quite variable, with Waco9 more virulent than Noco2, which is more virulent than Emco5. Such observations are usually interpreted in terms of variation in avirulence gene content. However, variation in host targets as well as in *Hpa* effector complement may also underpin quantitative differences in host susceptibility. *Hpa* isolate Emoy2 was reported as having the highest likelihood of producing high levels of sporulation in a study involving 96 *Arabidopsis* accessions, and isolate Emco5 the lowest [79]. Although *Hpa* virulence appears to depend on multiple virulence genes with weak effects, rather than a few genes with strong effects, some effectors, such as HaRxL62, 14, 44, 57 and 106, are particularly effective and will repay detailed mechanistic investigation in the future.

### HaRxLs that enhance *Pst* growth usually suppress callose deposition

Significantly, we found that most of the HaRxLs (77%) that increase *Pst*-LUX growth in Col-0 were also able to suppress *Pst*-ΔCEL-induced callose deposition. Conversely, those HaRxLs reducing *Pst*-LUX growth in Col-0 were generally unable to suppress callose deposition. Callose deposition is a late PTI response (though also associated with ETI). We speculate that HaRxLs may enhance *Pst*-LUX growth via additional PTI suppression, either alone or in conjunction with *Pst* effectors. The increased susceptibility to *Pst*-LUX observed using the EDV system was usually consistent with phenotypes of plant lines that constitutively express the corresponding HaRxLs (Figure 7). Moreover, seven of the transgenic lines also showed increased susceptibility to *Hpa* isolate Noco2. We infer that enhanced susceptibility results from suppression of host mechanisms that are active against diverse pathogens. The fact that they further elevate virulence conditioned by *Pst*-DC3000 effectors may reflect HaRxLs interference with targets that are not identical to those of *Pst*-DC3000, resulting in an additive effect.

### Infection with *Hpa*, and many *Hpa* effectors, suppress PTI

Assays using flg22-induced ROS or callose deposition on stable transgenic lines indicate that the main target of HaRxLs is PTI. All of the transgenic lines tested showed either reduced levels of flg22-induced ROS or callose deposition or both. The PAMP complement in *Hpa* is unknown, as are their receptors and downstream signal transduction pathways in *Arabidopsis*. Several molecules have been reported as oomycete PAMPs [17], [80] but their existence in *Hpa* is not known [9], [81]. We show that pre-elicitation of PTI by bacterial and fungal PAMPs impairs *Hpa* growth and reproduction, indicating that to infect, *Hpa* must counteract these host responses. Moreover, in host tissues where high numbers of haustoria are established, PTI responses are attenuated.

PTI involves multiple processes that can be attenuated by diverse pathogen effectors [76], [77], [78], [82]. Our data support the idea that the function of the majority of the effector proteins is to inhibit plant immunity [4], [83], [84]. For *Pst*, 13 out of 28 active effectors (12 belonging to *Pst*-DC3000) have been reported to suppress PTI [84], [85], [86]. Thus, ∼50% of this bacterial pathogen's effector repertoire targets PTI in one host. Importantly, this hemibiotroph can infect Solanaceae as well as Brassicaceae, so more effectors might emerge as PTI suppressors when other host species are studied. It has also been observed that 91% of *Pst*-DC3000 effectors, when delivered at high titers from Pf0-1, are able to suppress the HR induced by the bacterial effector HopA1 (from *P. syringae syringae*) in tobacco [38]. Since tobacco is a non-host for *Pst*-DC3000, this study again points to a high functional redundancy between effectors in suppressing HR. In oomycetes, experimental characterization of several RxLR effector genes suggests that many function to suppress host defenses [37], [50], [86]. Also, 3 out of 32 *P. infestans* RXLR candidate effectors were able to suppress PAMP- triggered programmed cell death (PCD) in *N. benthamiana*, while another 13 induced either non-specific or R-mediated PCD [87].

### HaRxLs are rarely avirulence determinants

We also identified HaRxLs that reduced *Pst*-LUX growth in the interaction with *Arabidopsis* accessions, and investigated whether they are new avirulence determinants (ATRs). Surprisingly, we observed that strong incompatibility caused by HaRxLs is rare. None was able to trigger macroscopic ETI when delivered *in planta* at high titer, as ATR13^Emco5^ did in Nd-0. Instead, several were identified that can reduce *Pst*-LUX growth in specific *Arabidopsis* accessions and four triggered micro HR-like lesions when delivered via *Pst*-ΔCEL. Since we tested only 64 HaRxLs from just one isolate, on only 12 host accessions, our survey was not exhaustive, and the anticipated ATR4 might not have been in our repertoire. Alternatively, the EDV assay may not be sensitive enough to detect new *Hpa* ATRs because these ATR-RPP recognitions are weaker than those already described with this system (ATR13-RPP13 or ATR1-RPP1). Conceivably, some ATRs might not carry an RxLR motif and therefore were not identified as candidate effectors in our bioinformatic analysis, as with the recently cloned ATR5 [88]. It also remains possible that either the sub-cellular localization or post-translational modifications of the EDV-delivered HaRxLs are not similar enough to their native form to be able to elicit ETI, although this has not been the case for ATR1 and ATR13 alleles [37], [50].

### Non-host resistance could involve a combination of recognized HaRxLs and ineffective HaRxLs

We also tested if the recognition or non-functionality of HaRxLs could be involved in non-host resistance to *Hpa* in *Brassica rapa* (turnip). We found that HaRxLs were “less effective” in turnip, but those HaRxLs that enhanced *Pst*-LUX growth in *B. rapa* also did so in *Arabidopsis*, suggesting conservation in their targets. Notably, three HaRxLs conferred reduced *Pst*-LUX and *Pst*-ΔCEL growth in *B. rapa*, but did not reduce growth in any *Arabidopsis* accession. Therefore, the inability of *Hpa* to grow in turnip might result not only from reduced “effectiveness” of the effector complement, but also from recognition in the “non-host” of a subset of effectors that are not recognized by most *Arabidopsis* accessions.

### Concluding observations

As with any screening protocol, this heterologous system has some limitations. For example, HaRxLs that require extensive post-translational modifications will not be correctly produced by a prokaryotic system. Also, the co-delivery of an HaRxLs with *c.a* 30 effectors from *Pst* DC3000 might alter the outcome of the assay if positive or negative interactions exist between them. This might explain some of the discrepancies we observed between results obtained with the EDV system and those generated by expressing the candidate effectors directly in the plant. A further potential limitation of the EDV system is that effectors required to elaborate a haustorium inside the host cell might not be revealed as promoting *Pst* growth by the assays we developed. Despite such limitations, most of the phenotypes observed with the EDV system were confirmed in the transgenic lines.

In conclusion, the EDV-based system has enabled the systematic analysis of the biological relevance of effector candidate proteins. The *Pst*-LUX and *Pst*-ΔCEL screens allowed the generation of a “ranking of effectors” that permitted the selection of highly interesting candidates as targets for subsequent mechanistic studies. Further detailed investigations of *Hpa* effectors will help reveal how *Hpa* alters host cellular processes to promote its growth and reproduction.

## Materials and Methods

### Bioinformatic identification of HaRxLs

Different versions of the genome of *Hpa* isolate Emoy2 (http://vmd.vbi.vt.edu; v3.0, v6.0 and v8.3.2) were translated in all 6 reading frames. ORFs from ATG to Stop codon were identified using FgenesH (www.softberry.com) and GETORF (http://emboss.sourceforge.net). Only sequences that encoding ≥100 aminoacids were considered. Secreted proteins were identified using SignalP v3.0 (score cutt-off >0.9), TargetP and PSort (www.psort.org) [89]. Proteins were considered as secreted if two out of three programs called the Signal Peptide as significant. HaRxLs were selected as fulfilling the following criteria: i) Signal Peptide (SP) length <30 amino acids, ii) RxLR-like motif (RxLR/Q, RxL) between 4 and 60 amino acids from the SP cleavage site, iii) predicted protein had >40 amino acids after the RxLR like motif. Redundancy in the ORF dataset was corrected using BlastP. Sequences with 100% identity and E<10^−5^ were clustered and simplified to the one with highest SP score. A sub-set of RxLR-EE proteins was identified carrying an acidic motif (EE, EER/G/D) [28] between 4 and 30 aminoacids from the RxLR like motif. The expression of HaRxLs was verified using the following resources: i) ESTs generated by Sanger sequencing and 454 sequencing from cDNA extracted from Emoy2 conidiospores [6], ii) Illumina sequence tags (SAGE using 3′ tags from 7 dpi infected tissue), iii) Illumina normalized/concatamerized cDNA (from 3 and 7 dpi infected tissue) (Ishaque et al., unpublished) and iv) RT-PCR with primers designed at 100 bp flanking the ORF sequence (Baxter et al., unpublished). The presence of the predicted and alternative ATGs and Stops Codons, as well as introns, was verified. Nucleotidic sequence polymorphisms on the HaRxLs accross seven *Hpa* isolates was assessed using either PCR products or *in silico* assemblies of Illumina short reads (Ishaque et al., unpublished). The HaRxLs were roughly classified as: No polymorphic (0 SNPs), Low (≥1 SNPs ≤5), Medium (≥6 SNPS ≤15) and High Polymorphic (>16 SNPs). To complete the characterization of the *Hpa* Emoy2 HaRxLs set, its sub-cellular localization (PSORTII) and presence of known conserved protein domains using Coil, Gene3D, HMMPfam, HMMSmart, HMMTigr, PFAM and Prosite was recorded. These data are available in Table S1 for the subset of HaRxLs cloned in this work.

### Cloning of HaRxLs

Selected HaRxLs were amplified from genomic DNA extracted from conidiospores of isolate Emoy2 using proofreading polymerase (Accuprime Pfx, Invitrogen) and standard PCR conditions. To generate the HaRxLs collection, the primers were designed to amplify from the signal peptide cleavage site or the RxLR (inclusive) until after the stop codon (3′ untranslated region, UTR). For cloning in pEDV3 or pEDV5 [37], the primers were designed to have SalI/ClaI and BamHI/BglII restriction sites at the 5′ and 3′ ends respectively. For cloning in pEDV6, a Gateway destination version of pEDV3, the sequence CACC was added at the 5′ of the Forward primer. PCR products were gel purified (Qiagen) and ligated (EDV3/5) or recombined in pENTRY-SD-D-TOPO/pDONR221 following the manufacturer's instructions and electroporated in *Escherichia coli* DH5∝. Gentamycin (EDV3/5) or kanamycin (pENTR/pDONR) resistant colonies were selected on plates and colony PCR performed with M13F and M13R primers. Colonies carrying the right size insert were selected for plasmid purification and sequencing. For EDV6, the correct inserts on pENTRY/pDONR vectors were recombined using Gateway LR clonase or LR clonase II enzyme mix (Invitrogen). The *in frame* fusion of vector-HA tag-HaRxL sequences were confirmed by sequencing with M13F and M13R primers. Plasmids were mobilized from *E.coli* DH5∝ to wild-type or mutant *Pst* strains by standard triparental matings using *E. coli* HB101 (pRK2013) as a helper strain. Bacterial growth in vitro was controlled at 12, 24, 32 and 44 hs post inoculation of 10 ml Kings B media with a dilution corresponding to 0.00001 OD of an overnight culture of each of the *Pst*-LUX clones harbouring a different HaRxL or control proteins (GFP, AvrRPS4^AAAA^). Three colonies per clone were assayed in different experiments. Growth was measured assessing turbidity at OD600 (for liquid cultures) or counting colonies of plated dilutions (in solid media). No significant differences in growth kinetics were observed for the *Pst*-LUX carrying HaRxLs regarding the clones carrying control proteins or the empty vector pEDV5.

### Bacterial strains

Bacterial strains used in this study include *E. coli* DH5∝, *Pseudomonas syringae* pv *tomato* DC3000 carrying the luxCDABE operon from *Photorhabdus luminescens* (*Pst*-LUX) [41], *Pseudomonas syringae* pv *tomato* DC3000 mutant ΔCEL [90], *Pseudomonas syringae* pv *tomato* DC3000 double mutant ΔavrPto/ΔavrPtoB [91], *Pseudomonas fluorescens* Pf0-1 carrying a functional TTSS [57] and *Agrobacterium tumefasciens* GV3101 (pMP90 RK). *E. coli*, and *Agrobacterium* were grown in low salt Luria-Bertani broth at 37°C (*E. coli*) or 28°C (*Agro*) using either liquid media or petri dishes. *Pseudomonas* strains were grown in either LB or King's B medium at 28°C in liquid media or petri dishes. Antibiotics concentrations (µg/ml) were as follows: Rifampicin 100, Kanamycin 50, Gentamycin 25, Spectinomycin 50, Chloramphenicol 50, Tetracycline 10, Carbenicillin 50.

### Plant materials and growth

*Arabidopsis* accessions used in this study were obtained from NASC. The *fls2-1* mutant was obtained from Cyril Zipfel and the *cerk1-1* mutant was a kind gift of JP Rathjen. Transgenic lines constitutively expressing HopU1 and HopAO1 were kindly provided by Jim Alfano. Turnip seeds (*Brassica rapa* cv Just Right) were purchased from Gurney's seeds (http://gurneys.com). Tobacco (*Nicotiana tabacum* cv petit Havana) and tomato (*Solanum lycopersicum* cv Moneymaker) seeds were obtained from John Innes Horticultural services. *Arabidopsis* plants were grown in Scotts and Levington F1 modular compost in controlled environment rooms under short day cycles (10 h/14 h day/night and 150–200 µE/m^2^s) at 22°C and 60% relative humidity and slightly watered every day from below. Tobacco, tomato and turnip plants were grown under similar conditions as *Arabidopsis* for 5 weeks post-germination. Plants expressing constitutively HaRxLs were generated by recombining the corresponding ORFs cloned in pDONR221 in the Gateway destination binary vector pB2GW7 [92] under the control of the CaMV 35S promoter. Constructs were transferred to *A. tumefaciens* strain GV3101 (pMP90 RK) [93] and transformed into *Arabidopsis* accession Col-0 by the floral dipping method. Primary transformants (T_1_) were selected on soil containing BASTA (Bayer CropScience, Wolfenbüttel, Germany) and self-pollinated. The progeny of the T_2_ generation was observed and 3∶1 (BASTA-resistant/BASTA-susceptible) segregating lines were taken further. Homozygous lines were selected by examining the BASTA resistance of T_3_ seedlings. Three independent transgenic lines per HaRxL (T4s) were analysed and for simplicity we present results for two.

### Pathogen growth and inoculations

Primary streaks of *Pst*-LUX complemented with the controls or HaRxLs were made from isolated colonies onto selective King's B plates and grown overnight at room temperature. Selected individual colonies were then spread with a sterile loop in solid KB plates and incubated overnight at room temperature to produce even bacterial lawns. Cells were scraped from plates with a sterile loop and suspended in 50 to 100 ml of 10 mM MgCl_2_ to a final OD_600_ of 1. Dilution series were made from these suspensions to: spray (OD_600_: 0.2) or infiltrate (OD_600_ = 0.001) *Arabidopsis* plants, or to infiltrate tobacco (OD_600_ = 0.01), tomato (OD_600_ = 0.001) or turnip (OD_600_ = 0.001) plants. For tobacco and turnip, leaf panels of the third- to fifth-oldest leaves of were infiltrated by pricking the leaves with a dissecting needle and infiltrating with a blunt syringe. pEDV-HaRxLs were compared with controls on the same leaf. For tomato, leaflets of the third and fourth most recently expanded leaves were used. Concentrations of other bacterial pathogens used in this work are stated on the corresponding figure legends or other sections of M and M. *Hpa* isolates Emoy2 and Noco2 were maintained in compatible host accessions and inoculated onto 2-week-old plants at 1 or 5×10^4^ conidiospores/ml. After infection, plants were covered with a transparent lid to maintain high humidity (90–100%) conditions in a growth chamber at 16°C for 7 days in short day (10 h/14 h day/night) cycle. To increase the ratio pathogen/host biomass for gene expression analysis, plants were sprayed with the conidiospores solution (or water as control) were kept uncovered in low humidity (60%).

### EDV-*Pst* LUX screening method

*Arabidopsis* plants of all 12 different accessions were grown in arrays of five individual cells and shuffled randomly before inoculation. Five four-week-old plants of each accession were sprayed with *Pst-*LUX bacterial suspensions (2×10^8^ cfu/ml, 0.03% (v/v) Silwet L-77) carrying pEDVs encoding for control or HaRxL proteins. Spraying was done using an airbursh system attached to a compressor (GS, model AS18). About 3 mls of bacterial suspension was used *per* five plants at a pressure of 10–12 psi. Sprayed plants were kept under a transparent lid to keep high humidity conditions. At three days post-spraying, sets of whole five plants were placed in an ultra low light CCD camera (Photek, www.photek.com). Photons emitted per second were scored per plant and referred to the whole plant fresh weight to account for the foliar area. The average level of Photon Counts per Second per gram of Fresh Weight was obtained for each *Pst*-LUX EDV-delivered protein and the ratio versus the control was scored on each accession (Figure 2). To verify the correspondence between increase on photon emission and bacterial population growth, leaf discs were sampled from the five plants sprayed to generate at least 6 technical replicates. The leaves were surface sterilized (30 s in 70% ethanol, then 30 s sterile distilled water). Traditional growth curve assays were performed as described [94].

### Gene expression analysis

Total RNA was extracted from three-week-old HaRxL overexpressing lines (T4 generation) using the RNeasy Plant Mini Kit (Qiagen, Hilden, Germany). 1 µg DNAse-treated total RNA was reverse transcribed using the QuantiTect Reverse Transcription Kit (Qiagen). For semi-quantitative RT-PCR 1 µl of cDNA was used per reaction and amplified with an initial denaturation step at 95°C for 3 minutes followed by 23 cycles with the following conditions: 45 s at 94°C, 30 s at 55°C and 30 s at 72°C. In a last cycle a final elongation step at 72°C for 5 min was added. PCR products were separated on 1.5% TAE agarose gels. To control the equal amount of cDNA in every reaction the Actin2 gene (At3g18780) was used. The specificity of the primers for amplification of the HaRxLs transcripts was tested on pDONR221 clones harboring the corresponding HaRxLs. In case of control RT-PCRs on *Arabidopsis* Col-0 wild type plants cDNA, no unspecific amplification using the HaRxLs' primers was observed. The sequences of primers used in this study are available on request.

### Measurement of Reactive Oxygen Species (ROS) generation

ROS released by the leaf tissue in response to flg22 was measured using a chemiluminescent assay [95]. Briefly, leaf discs (0.38 cm^2^) from Col-0, *fls2-1* mutant, transgenic lines expressing a given 35S-HaRxL lines, and control lines were sampled. At least 24 leaf discs from four five-week-old plants per accession/line were sampled and floated for 14–16 h in sterile distilled water in a 96 flat-bootom white multiwell plate (Greiner Bio-One) kept in the dark. ROS production in response to flg22 was measured by replacing the water by 100 ul of a working solution of Luminol (34 µg/ml final concentration, Sigma), Horseradish Peroxidase (20 µg/ml final concentration, Sigma) and flg22 (100 nM final concentration, Peptron, South Korea). The plate was immediately introduced in a Luminometer (Varioskan, Thermo Scientific) and photon counts were recorded every 40 seconds for at least 40 minutes.

### Callose and Trypan Blue Stainings and microscopic analysis

Leaves of five-week-old plants were hand-infiltrated at the bottom of the leaf area with 5×10^7^ cfu/ml (OD_600_ = 0.05) *Pst-*ΔCEL suspensions. Bacteria complemented with control or HaRxL proteins were infiltrated in different plants of the same set. Twenty leaf samples were taken 12 to 14 h after inoculation at the top of the infiltrated area to avoid visualization of mechanical damage induced callose. Leaf discs were cleared 2 times (1 h) in 96% ethanol and then re-hydrated in ethanol/water series (70%, 50%, 30%) per 30 min each. Leaf disc were floated in water 1 h and then transferred to a solution of 0.01% (w/v) Aniline Blue (Gurr, BHD, England) in K_2_HPO_4_ Buffer (150 mM pH 9.5) for 1 h. The samples were then transferred to Glycerol 60% (v/v) and mounted for observation with a Leica DM R fluorescence microscope using UV light and I3 filter (A4-UV). Pictures were taken with a Leica DFC 300 FX Digital Camera. Images were analyzed and callose dots quantified using Image J software and an *in house* written Macro. The same imaging system was used to visualize *Hpa* infection structures after staining with lactophenol trypan blue. The number of conidiophores *per* leaf was scored by manually scanning the abaxial and adaxial surfaces of each leaf on at least four true leaves of five-ten plants per accession/transgenic line analyzed.

### Ion leakage measurements

Five-week-old plants were infiltrated with 1×10^8^ cfu/ml (OD_600_ = 0.1) *P. fluorescens* Pf0-1 carrying pVSP61-AvrRPM1. To be able to detect HR symptoms the bacteria had to be grown in plates. Leaf discs were collected right after infiltration using a cork borer number 4. Twenty-four leaf discs were collected per transgenic line and shacked in falcon tubes with 45 ml distilled water for 1 h at room temperature. Four leaf discs were transferred multi-well plates with 2 ml distilled water. The level of ion leakage caused by AvrRPM1 recognition by RPM1 in the Col-0 background was detected as an increment in conductivity. This was measured with a Conductivity meter (Horiba Twin B-173) every 60 min over 14 h on at least 6 technical replicates per transgenic line.

### Secretion assay and protein analysis

To verify the accumulation proteins corresponding to the AvrRPS4-effector fusions in *Pseudomonas* and its secretion by the TTSS, the strains complemented with *Hpa* HaRxLs were grown overnight at 28°C in 25 ml liquid LB media to a final OD_600_ = 0.3, centrifuged, washed twice with 10 mM MgCl_2_ and re-suspended in 20 ml minimal media (*hrp*-inducing minimal medium, MM) [50 mM potassium phosphate buffer, 7.6 mM (NH_4_) 2SO_4_, 1.7 mM MgCl_2_, 1.7 mM NaCl, pH 6.0, with 20 mM glucose added]. The cultures were incubated at 22°C at 200 rpm on a rotator shaker to a final OD_600_ of 0.5. The pellet and supernatant fractions were separated by centrifugation at 5,200× *g* for 15 min at 4°C. The pellets were re-suspended in 300 ul of bacterial protein extraction buffer [100 mM NaCl, 25 mM Tris-HCl pH 8, 10% glycerol, 10 mM DTT and 1× protease inhibitors cocktail (Complete EDTA-free tablets, Roche)], sonicated 3 times (10 s), and centrifuged at 12.000 rpm for 5 minutes. Then 100 µl of supernatant were taken and 25 µl of SDS loading buffer were added. Samples were boiled for 5 minutes at 96°C before loading. The supernatant of the 20 ml MM culture was filtered through a 0.2 µm pore filter and concentrated using centricon YM-10 columns (Amicon Bioseparation) by sequential centrifugations of 30 min at 5000 g at 4°C. The process was repeated several times. Proteins in the final 2 ml of concentrated supernatant were precipitated using Strataclean beads (Stratagene). Five to ten µl of beads were used per ml of supernatant; incubated 10 min at 4°C inverting gently, centrifuged at 2000 g for 2 min and re-suspended in 25 ul Laemmli buffer containing 0.1 M NaOH. Samples were boiled for 5 minutes at 96°C and centrifuged at 12.000 rpm for 2 minutes before loading. The pellet fraction (15 µl) and the culture fluid fraction (25 µl) were analyzed by SDS–PAGE, electro blotted onto PVDF membrane (Bio-Rad), and probed with horseradish peroxidase- conjugated high affinity anti–HA antibody (Roche) and re-probed with anti-NPTII antibody (Upstate). Bands were visualized using PICO kit (Thermo Scientific) and imaged with Kodak scientific imaging film.

## Supporting Information

Figure S1 **Verification of the functionality of the TTSS of** ***Pst*** **-LUX clones expressing HaRxLs.** (A) Immunoblot showing the accumulation of AvrRPS4 _1–136_-HA-HaRxL fusions on the bacterial pellet (left panel) and its secretion into TTSS-inducing minimal media (right panel, supernatant). Approximated molecular weights in KDa are shown on the left of each panel. (B) Four-week old tobacco (*N. tabacum* cv. *petit havana*) leaves were infiltrated with *Pst*-LUX clones carrying either control proteins (YFP, AvrRPS4-^AAAA^), or HaRxLs at OD_600_ = 0.01. *Pst*-hrcC and *Pst*-hrcC-LUX strains were included as positive controls for TTSS impairment. Symptoms of HR cell death were screened at 2,3 and 4 dpi. Picture was taken at 2 dpi. (C) Five-week old tomato (*Solanum lycopersicum* cv. *moneymaker*) leaflets syringe infiltrated with OD_600_ = 0.001 of different *Pst*-LUX clones expressing HaRxLs or controls (YFP). Leaflets were detached and imaged at 3 dpi with a Photek camera to detect bioluminescence.
(TIF)

Figure S2 **Correlation between** ***Pst*** **-LUX bioluminescence and its growth** ***in planta*** **.** (A), (C) Five-week-old plants of the indicated *Arabidopsis* accessions were spray-inoculated at OD_600_ = 0.2 with *Pst*-LUX delivering the indicated *Hpa* effector or control proteins. At 3 dpi, five whole plants per treatment were imaged using a Photek camera to record photons counts per second. Bars illustrate the average photon counts per gram of plant fresh ± SD. (a) p value of T-test assuming unequal variances <0.05, (b) p<0.01. (B), (D) Twenty-four leaf discs obtained from the above mentioned plants were excised and used to determine the number of bacteria per leaf area, showed in Log_10_ scale. Bars indicate the average ± SD of six technical replicates. One-way ANOVA test was applied with (a) p<0.05, (b) p<0.01.
(TIF)

Figure S3 **Behavior of** ***Pst-*** **LUX delivering HaRxLs assessed via growth curves.** Histograms illustrate the changes in growth levels (measured as colony forming units –CFU-) of *Pst*-LUX strains delivering the indicated HaRxLs, compared to control strains, on different *Arabidopsis* accessions.
(TIF)

Figure S4 **Pattern of HaRxLs induced changes in** ***Pst*** **-LUX virulence plotted per** ***Arabidopsis*** **accession.** Bars indicate the number of HaRxLs that enhanced (green), decreased (red) or did not changed (black) the growth of *Pst*-LUX on each *Arabidopsis* accession tested. The outcome of the interaction of the *Hpa* isolate Emoy2 with each accession is indicated as Susceptible (S) or Resistant (R). Known and predicted ATR/RPP interactions are described. (?) indicate putative/unknown ATR/RPP genes.
(TIF)

Figure S5 **Semi-quantitative RT-PCR applied to RNA extracted from the stable transgenic lines generated and tested in this paper.** Expression levels of each HaRxL were tested in two independent homozygous transgenic lines (1,2). For comparison, the expression of the *Arabidopsis* housekeeping gene actin (Act2) is shown.
(TIF)

Figure S6 ***Arabidopsis*** **Col-0 plants expressing constitutively HaRxLs support enhanced growth of** ***Pst*** **-LUX.** Five four-week-old plants of two independent transgenic lines expressing the corresponding *Hpa* candidate effector and one line per control protein, were sprayed at OD_600_ = 0.2 with *Pst*-LUX. At 3 dpi, photon counts per plant were measured, as well as plant's fresh weight. Bars represent means of 5 replicates ±2× Standard Errors (SE). (a) p value of T-test assuming unequal variances <0.05; (b) p<0.01. This experiment was repeated three times with similar results.
(TIF)

Figure S7 **Constitutive expression of four different HaRxLs caused a mild reduction on the levels of ion leakage triggered by AvrRPM1 recognition.** Five-week-old plants were hand infiltrated with Pf0-1 delivering *P. maculicola* AvrRPM1 at OD_600_ = 0.1. Twenty-four leaf discs were sampled from four infiltrated transgenic plants per line per HaRxL in each of five different experiments. At least six technical replicates were done per line. Conductivity was measured in the water were discs were floating, as an indication of ion leakage into the media. Measurements were taken every hour until the peak of ion leakage was detected in the wild type and control lines (around 14 hours post-infiltration). Bars correspond to the average ±2× SE of the maximum level of ion leakage observed for each line expressed as a percentage of the value displayed by Col-0 wild type (100%). Values take into account averaged results of 5 different experiments. (a) p value<0.05 of two tailed Z-test.
(TIF)

Figure S8 **Fewer HaRxLs can alter the growth of** ***Pst*** **-LUX in the** ***Hpa*** **-non-host** ***Brassica rapa*** **compared to** ***Arabidopsis*** **.** Columns show the distribution of the number of candidate effectors that enhance or decrease *Pst*-LUX virulence in the non-host *B. rapa* compared to one accession (Col-0) and the average results of the screening in the set of twelve accessions of the host *Arabidopsis*.
(TIF)

Table S1 **Names and sequences of** ***Hyaloperonospora arabidopsidis*** **isolate Emoy2 HaRxLs candidates cloned.** (a) Nucleotide sequence predicted in genome versions of the Hpa Emoy2 race (V3.0 to V6.0) generated previously to the published one (v8.3.2; Baxter and Tripathy et al., 2010). (b) Translation of the predicted nucleotide sequence in Hpa Emoy Genome v8.3.2. (c) Nucleotidic sequence cloned in pENTRY/pDONR vectors. The clones were amplified from the Signal Peptide cleavage until the stop codon, adding a Methionine (M) at the N-terminal, unless stated otherwise. (d) Sequence cloned in pEDV vectors. When pEDV6 was used, sequences were identical to the pENTRY donors. For clones in pEDV3/5 (non-Gateway versions) no pENTRY clones were generated. (e) Comparison of sequence similarity of the pEDV clone with the predicted nucleotide sequence on the Emoy2 genome (Column G vs Column C). (f) Comparison of sequence similarity of the pEDV clone translated aminoacid sequence with the predicted one the Emoy2 genome (Column H vs Column D). (g) Level of Polymorphism of candidate RxLR effectors predicted by “*in silico*” comparison of the *Hpa* Emoy2 race genome (v8.3.2, v6.0, v3.0) with the genomes of seven Hpa races sequenced via illumina short reads. The number of SNPs predicted is indicated between brakets. We classified them as No polymorphic (0 SNPs), Low (≥1 SNPs ≤5), Medium (≥6 SNPS ≤15) and High Polymorphic (>16 SNPs). (h) Level of Expression of candidate RxLRs was tested using different expression libraries, and categorized as: 1- Sanger ESTs obtained from Spores, 2- 454 ESTs generated from infected tissue at 3 days post-inoculation (dpi), 3- Illumina short reads generated from infected tissue at 3 and 7 dpi. (i) Predicted subcellular localization using Wolf-PSORT (of sequences after signal peptide cleavage site). The localization stated corresponds to the ones consistently found using both Plant and Fungi databases. The score numbers representing the number of closest neighbours in each alignment are represented in brakets. (+) Cloned from RxLR onwards, without M. (*) Cloned from RxLR onwards, X or L replaced by M. (°) Cloned from SP cleavage site onwards, with M introduced. ND Not determined. (1) There are two sequences highly similar in Emoy2 genome. The paralog cloned here is not assembled in the published Emoy2 genome (v8.3.2). They differ only in three aminoacids (EGMIW×KNMIR). (2) Putative gene family. The paralog cloned is not the one assembled in V8.3.2 of the genome. (3) The cloned sequence not assembled in v8.3.2. It is highly similar except for the C-terminal end. Putative paralogs. (4) Putative gene family. The paralog cloned is not assembled in v8.3.2 of the genome. (5) There are two paralog sequences in Emoy2 genome. Only HaRxL45 is assembled in v8.3.2. (6) These RxLR candidates are not assembled in the V8.3.2 of the genome, but they were predicted in previous versions of it (v3.0 and v6.0). (7) The sequence of this clone has an internal deletion of 20 aminoacids regarding the sequence predicted in v8.3.2 of the genome. Putative paralog. (8) NC1,2,3,4: Negative controls.
(XLS)

Table S2 **Effect of 64 HaRxLs on** ***Pst*** **-LUX bioluminescence tested across 12** ***Arabidopsis*** **accessions.** (a) *Pst-LUX* complemented with the corresponding candidate HaRxL or control was infiltrated in leaves of *Nicotiana tabacum cv. Petit Havana*. Development of hypersensitive response (HR) cell death was evaluated at 2 days post-infiltration. Representative picture on Figure S1B. (b) *Pst-LUX* complemented with the corresponding candidate HaRxL or control was infiltrated in leaflets of *Lycopersicum esculentum cv. Moneymaker*. Disease sympthoms were determined between 3 and 5 dpi. Presence of the bacteria on the lessions was confirmed imaging Pst-LUX bioluminescence. Representative picture on Figure S1 C. (c) Values in the body of the table correspond to the ratio of Pst-LUX bioluminescence between a Pst-LUX clone delivering a given HaRxL candidate effector and a *Pst-LUX* clone delivering a control protein. The bioluminescence counts per second (CPS) per mg of plant fresh weight (FW) were averaged for five plants per Arabidopsis accession per experiment. In summary: Value = [X (CPS/FW) effector/X (CPS/FW) control] (d) Summary of the *Hpa* candidate effector's effect per ecotype. This conclusion is based on the reproducibility of a given trend of change (2 out of 3 experiments at least) and the statistical significance of the change regarding the control on each individual experiment. T-test p values assuming unequal variances are indicated as follows: >0,01 dark grey boxes, >0,05 light grey boxes. Mathematical symbols indicate increased (+), decreased (−) or no change ( = ) in bioluminescence/FW regarding control. (e) Number of *Arabidopsis* accessions on wich *Pst-LUX* bioluminescence activity increased (+), decreased (−) or did not change ( = ) when delivering a given HaRxL candidate effector. (f) Scoring the bioluminescence activity of *Pst-LUX* delivering a given HaRxL candidate effector in *Brassica rapa cv Just Right* (Turnip) leaves. Symbols as in (e). (g) Negative controls.
(XLS)

Table S3 **Comparison of photon counts** ***versus*** **colony forming units for** ***Pst*** **-LUX.** Results obtained delivering 13 *Hpa* candidate effectors via EDV by *Pst*-LUX in different *Arabidopsis* accessions. Numbers in the body of the table correspond to the ratio between *Pst*-LUX delivering the indicated effector and the control *Pst*-LUX clone delivering YFP. For the spray inoculation, the quantization of the bacterial bioluminescence (Photon Counts) and colony forming units (CFUs) is displayed for two to 3 replicate experiments. Experiments displaying the results obtained when the bacteria are introduced in the plant by syringe infiltration are shown for comparison. For the spray inoculation experiments, bacterial growth was scored at 3 dpi using five plants to record *Pst*-LUX bioluminescence. The same plants were ground to determine bacterial growth by plating in selective media and CFUs. Experiments in parallel were done inoculating the bacteria at OD_600_: 0.001 by syringe infiltration. Numbers highlighted in bold indicate T-test p value<0.05. (*) Result differs from the one obtained with the EDV screen (see Table S2). (a) Values of photon counts correspond to the ratio of counts per second (CPS)/fresh weight (FW) in grams of *Pst*-LUX clones delivering via EDV the stated *Hpa* candidate effectors versus CPS/FW of the YFP or AvrRPS4^AAAA^ control. (b) Values of CFUs correspond to the ratio colony forming units (CFUs)/FW of *Pst*-LUX clones delivering via EDV the stated *Hpa* candidate effectors versus CFUs/FW of the YFP control or AvrRPS4^AAAA^ control. (c) The concordance between the Photon counts and CFUs ratios is indicated.
(DOC)

Table S4 **Putatively recognized** ***Hpa*** **candidate effectors and HR-like cell death.** The table displays the number of leaves showing HR-like micro-lesions when Trypan blue-stained 24 h post-infiltration with *Pst*-ΔCEL (OD_600_ = 0.01) or Pf0-1 (OD_600_ = 0.1) delivering the indicated *Hpa* RXLR effector candidates in the corresponding *Arabidopsis* accessions. Numbers in parenthesis correspond to Pf0-1 data. Twenty-four leaves were infiltrated per candidate per bacterium. This experiment was repeated twice for each vehicle bacterium, with similar results. Values significantly different from negative controls (NC1 or NC2) across replicate experiments are indicated in bold (p value T-test<0.05).
(DOC)
