# Supplementary figures and images for: Multiple Candidate Effectors from the Oomycete Pathogen Hyaloperonospora arabidopsidis Suppress Host Plant Immunity
Source: PLoS Pathog. 2011 Nov 3;7(11):e1002348. doi: 10.1371/journal.ppat.1002348 (PMC3207932; doi:10.1371/journal.ppat.1002348)

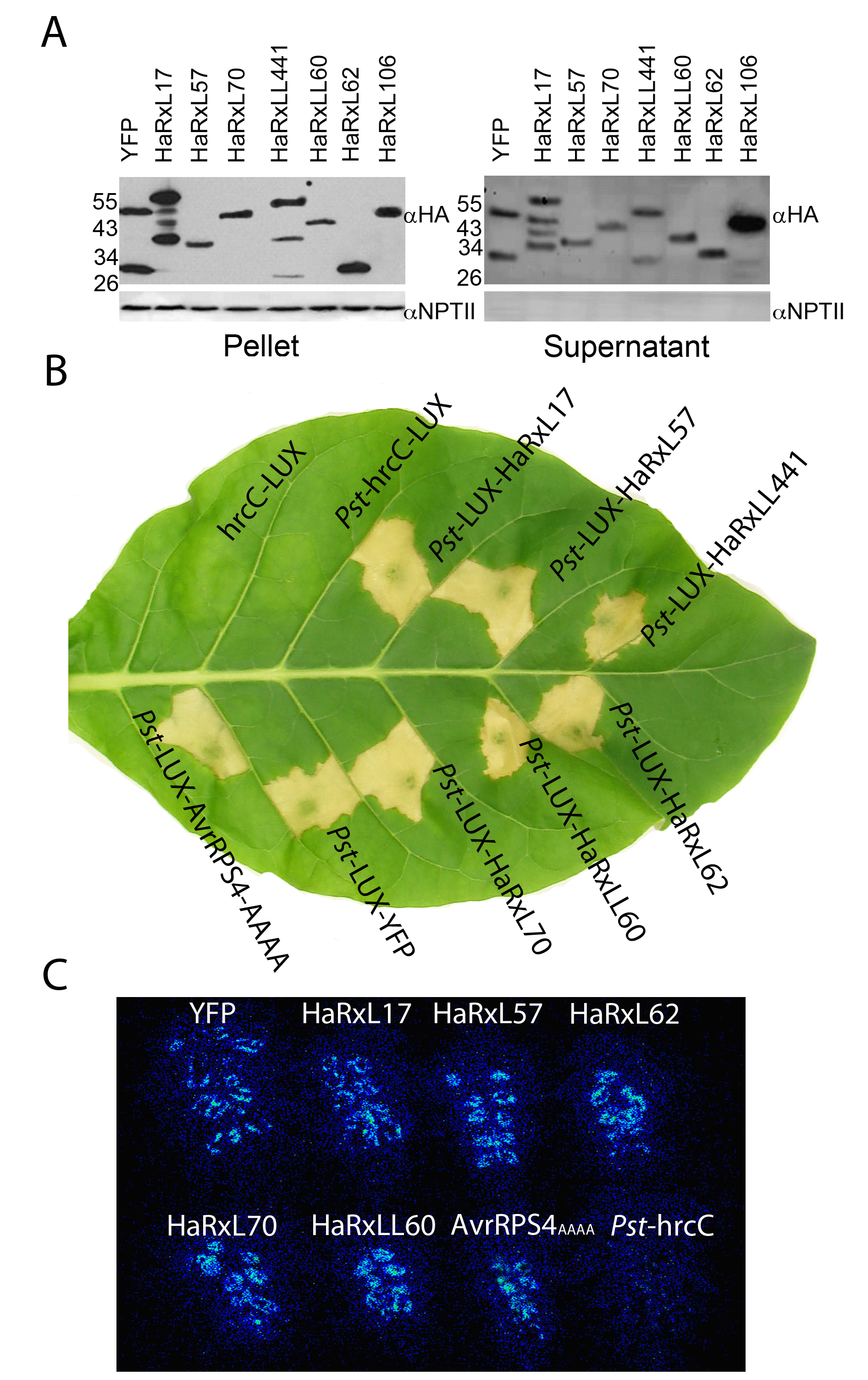

Supplement: Figure S1 — Verification of the functionality of the TTSS of Pst -LUX clones expressing HaRxLs. (A) Immunoblot showing the accumulation of AvrRPS4 1–136-HA-HaRxL fusions on the bacterial pellet (left panel) and its secretion into TTSS-inducing minimal media (right panel, supernatant). Approximated molecular weights in KDa are shown on the left of each panel. (B) Four-week old tobacco (N. tabacum cv. petit havana) leaves were infiltrated with Pst-LUX clones carrying either control proteins (YFP, AvrRPS4-AAAA), or HaRxLs at OD600 = 0.01. Pst-hrcC and Pst-hrcC-LUX strains were included as positive controls for TTSS impairment. Symptoms of HR cell death were screened at 2,3 and 4 dpi. Picture was taken at 2 dpi. (C) Five-week old tomato (Solanum lycopersicum cv. moneymaker) leaflets syringe infiltrated with OD600 = 0.001 of different Pst-LUX clones expressing HaRxLs or controls (YFP). Leaflets were detached and imaged at 3 dpi with a Photek camera to detect bioluminescence. (TIF) [file ppat.1002348.s001.tif]

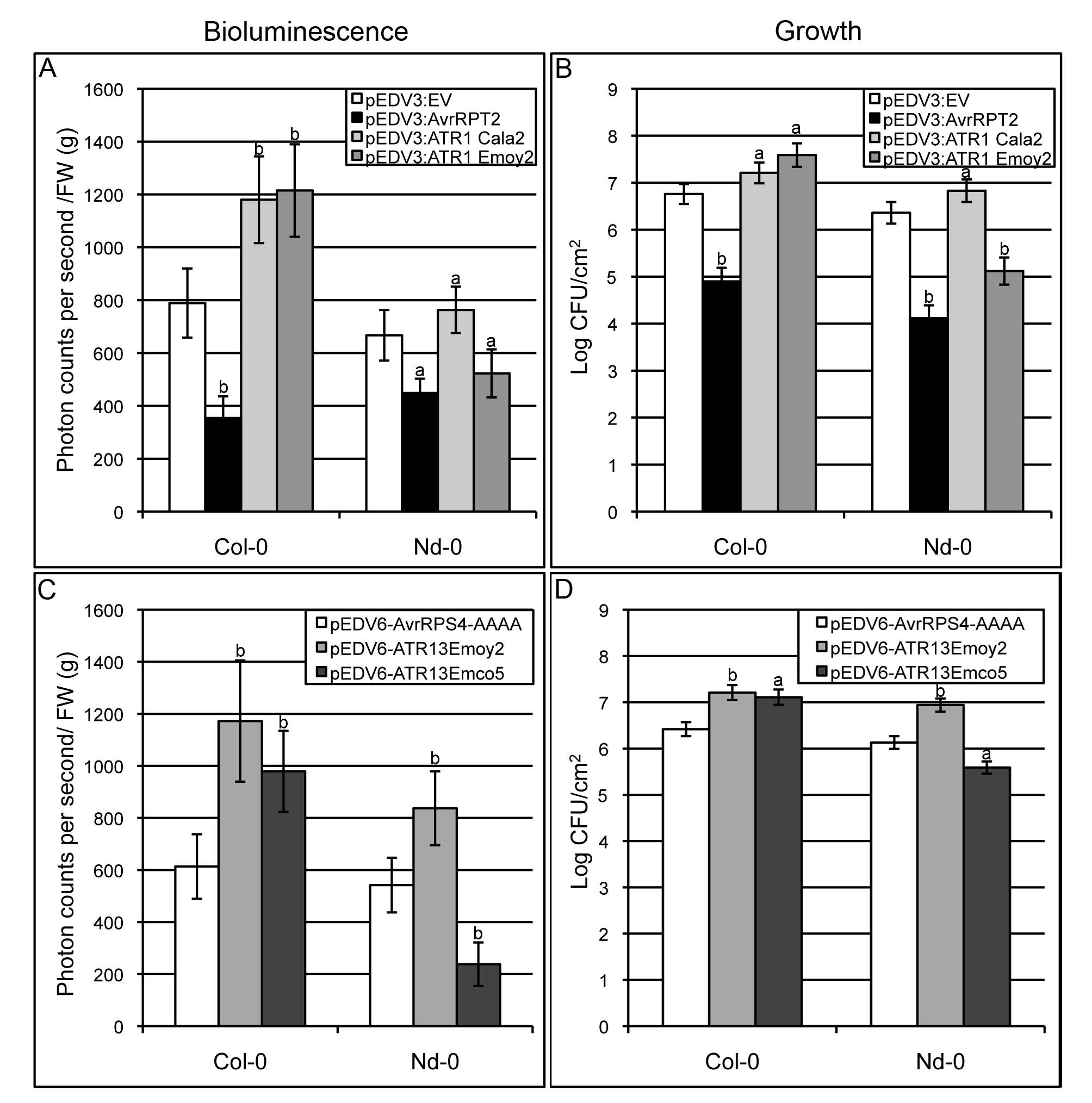

Supplement: Figure S2 — Correlation between Pst -LUX bioluminescence and its growth in planta . (A), (C) Five-week-old plants of the indicated Arabidopsis accessions were spray-inoculated at OD600 = 0.2 with Pst-LUX delivering the indicated Hpa effector or control proteins. At 3 dpi, five whole plants per treatment were imaged using a Photek camera to record photons counts per second. Bars illustrate the average photon counts per gram of plant fresh ± SD. (a) p value of T-test assuming unequal variances <0.05, (b) p<0.01. (B), (D) Twenty-four leaf discs obtained from the above mentioned plants were excised and used to determine the number of bacteria per leaf area, showed in Log10 scale. Bars indicate the average ± SD of six technical replicates. One-way ANOVA test was applied with (a) p<0.05, (b) p<0.01. (TIF) [file ppat.1002348.s002.tif]

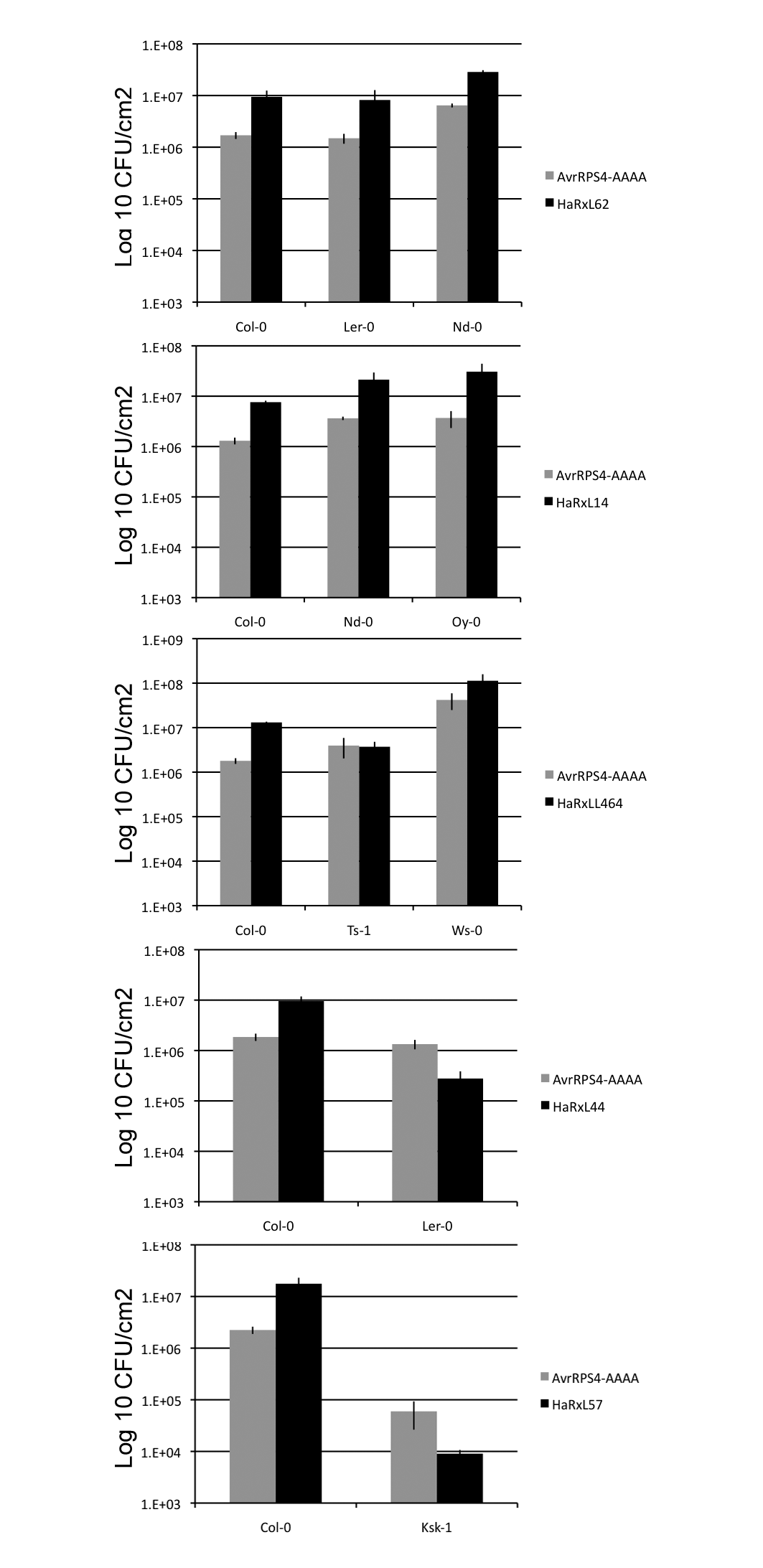

Supplement: Figure S3 — Behavior of Pst- LUX delivering HaRxLs assessed via growth curves. Histograms illustrate the changes in growth levels (measured as colony forming units –CFU-) of Pst-LUX strains delivering the indicated HaRxLs, compared to control strains, on different Arabidopsis accessions. (TIF) [file ppat.1002348.s003.tif]

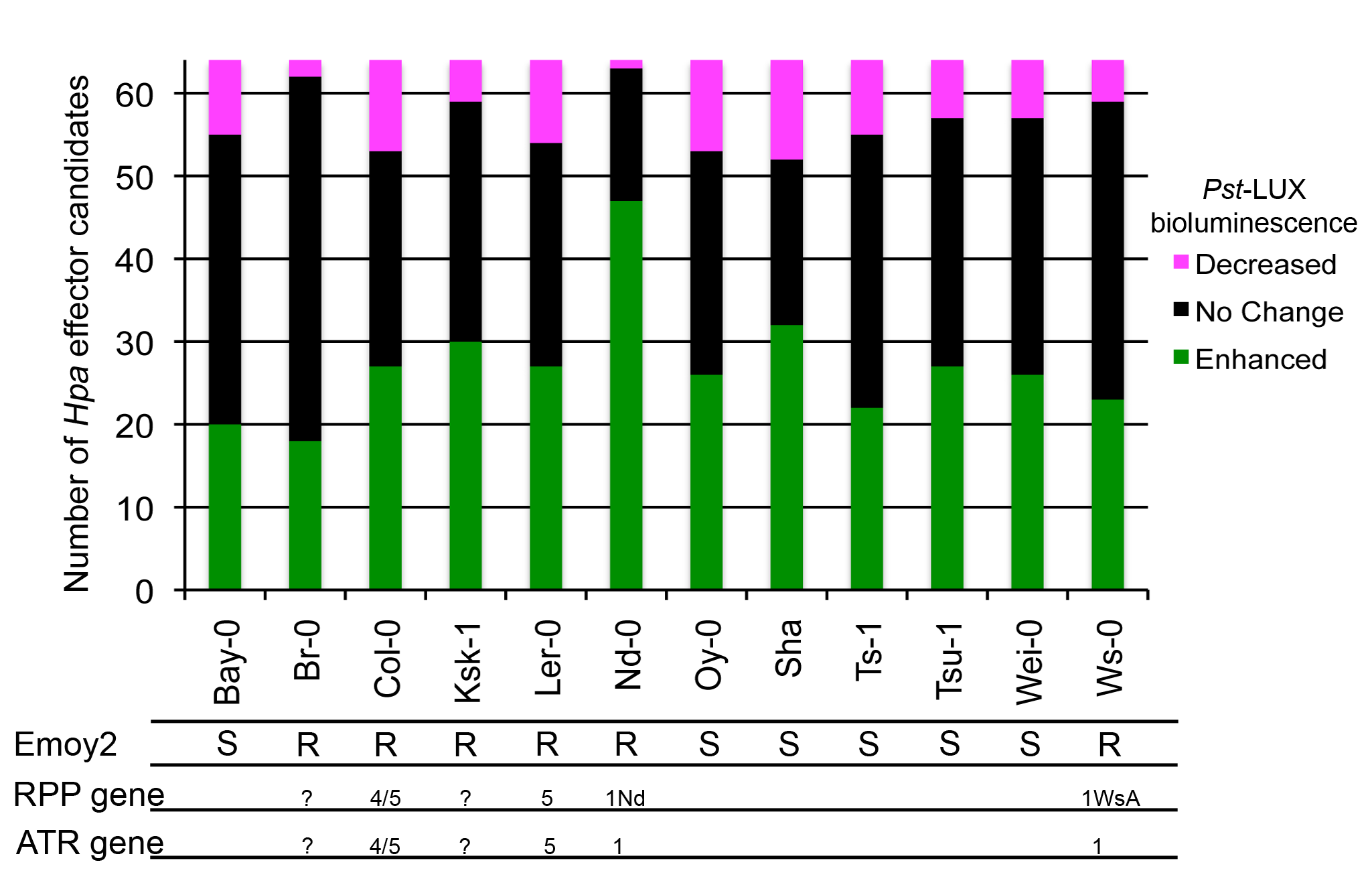

Supplement: Figure S4 — Pattern of HaRxLs induced changes in Pst -LUX virulence plotted per Arabidopsis accession. Bars indicate the number of HaRxLs that enhanced (green), decreased (red) or did not changed (black) the growth of Pst-LUX on each Arabidopsis accession tested. The outcome of the interaction of the Hpa isolate Emoy2 with each accession is indicated as Susceptible (S) or Resistant (R). Known and predicted ATR/RPP interactions are described. (?) indicate putative/unknown ATR/RPP genes. (TIF) [file ppat.1002348.s004.tif]

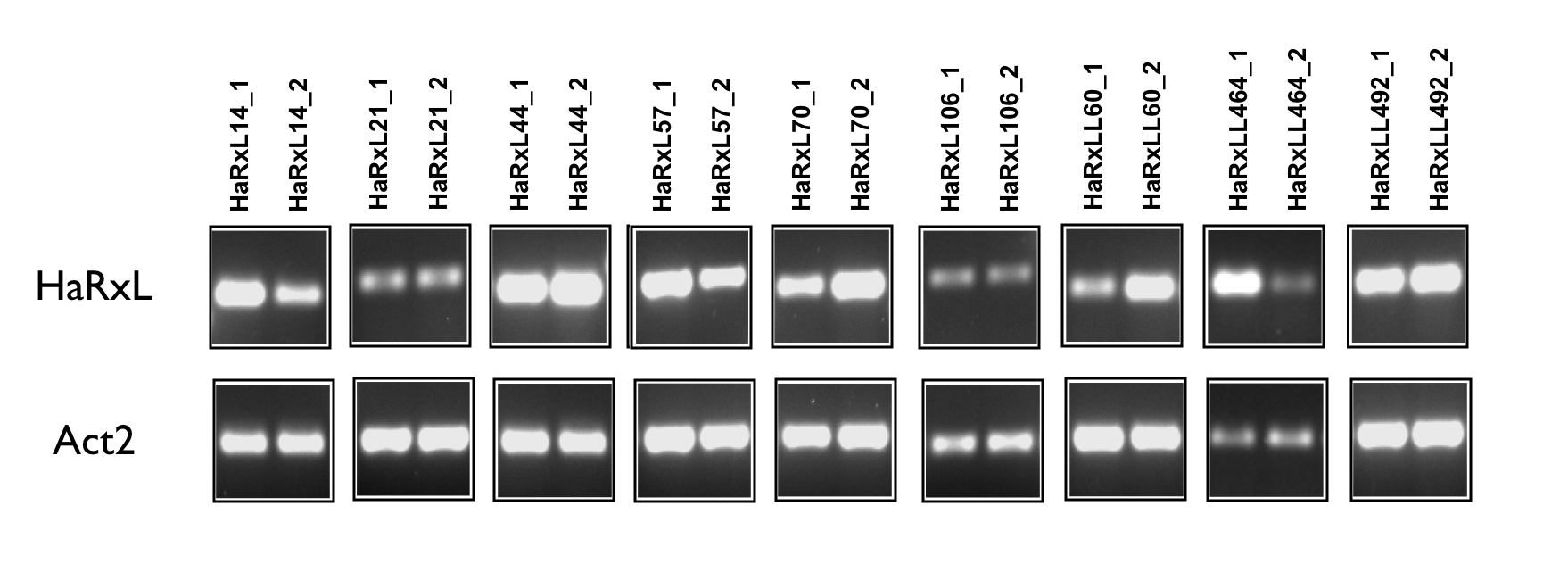

Supplement: Figure S5 — Semi-quantitative RT-PCR applied to RNA extracted from the stable transgenic lines generated and tested in this paper. Expression levels of each HaRxL were tested in two independent homozygous transgenic lines (1,2). For comparison, the expression of the Arabidopsis housekeeping gene actin (Act2) is shown. (TIF) [file ppat.1002348.s005.tif]

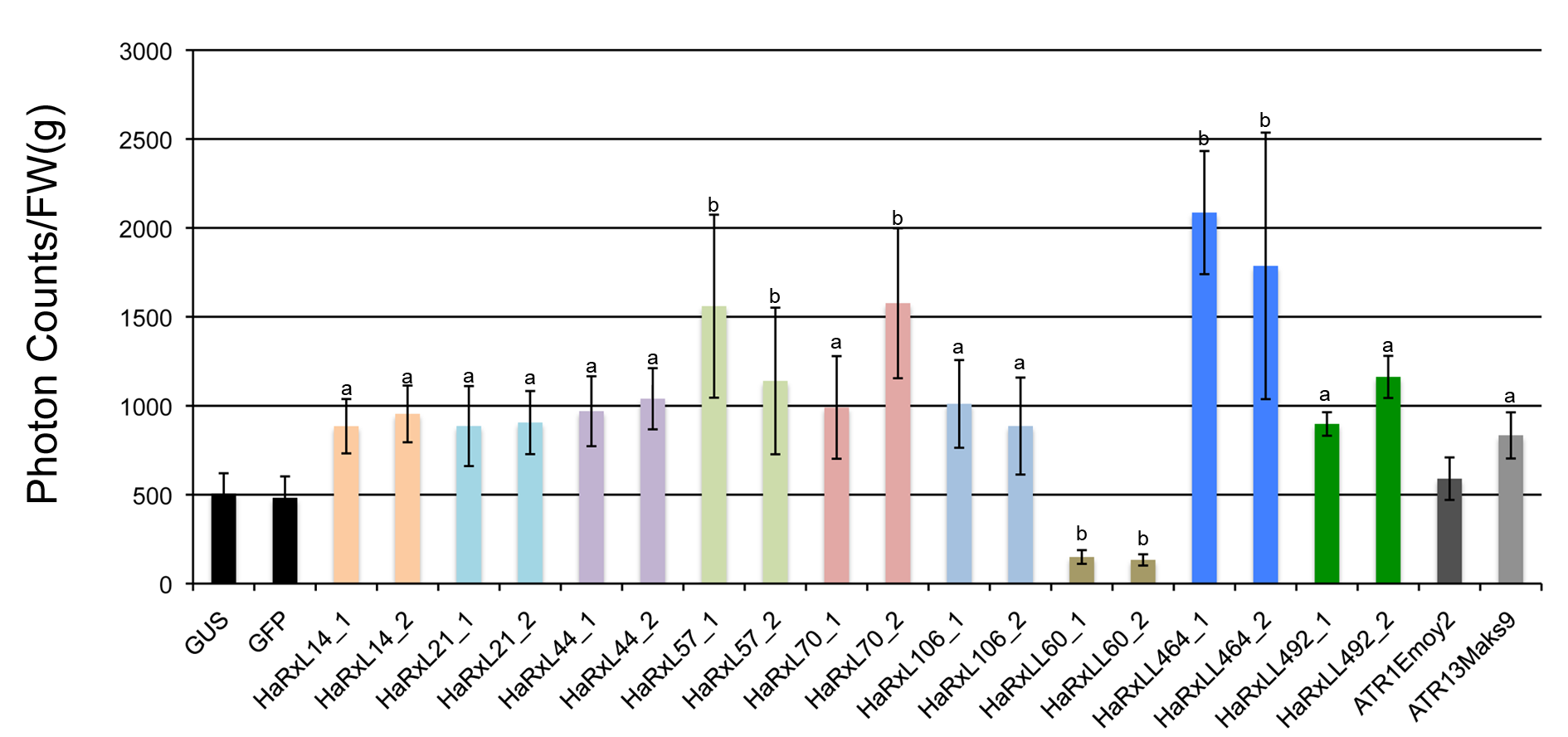

Supplement: Figure S6 — Arabidopsis Col-0 plants expressing constitutively HaRxLs support enhanced growth of Pst -LUX. Five four-week-old plants of two independent transgenic lines expressing the corresponding Hpa candidate effector and one line per control protein, were sprayed at OD600 = 0.2 with Pst-LUX. At 3 dpi, photon counts per plant were measured, as well as plant's fresh weight. Bars represent means of 5 replicates ±2× Standard Errors (SE). (a) p value of T-test assuming unequal variances <0.05; (b) p<0.01. This experiment was repeated three times with similar results. (TIF) [file ppat.1002348.s006.tif]

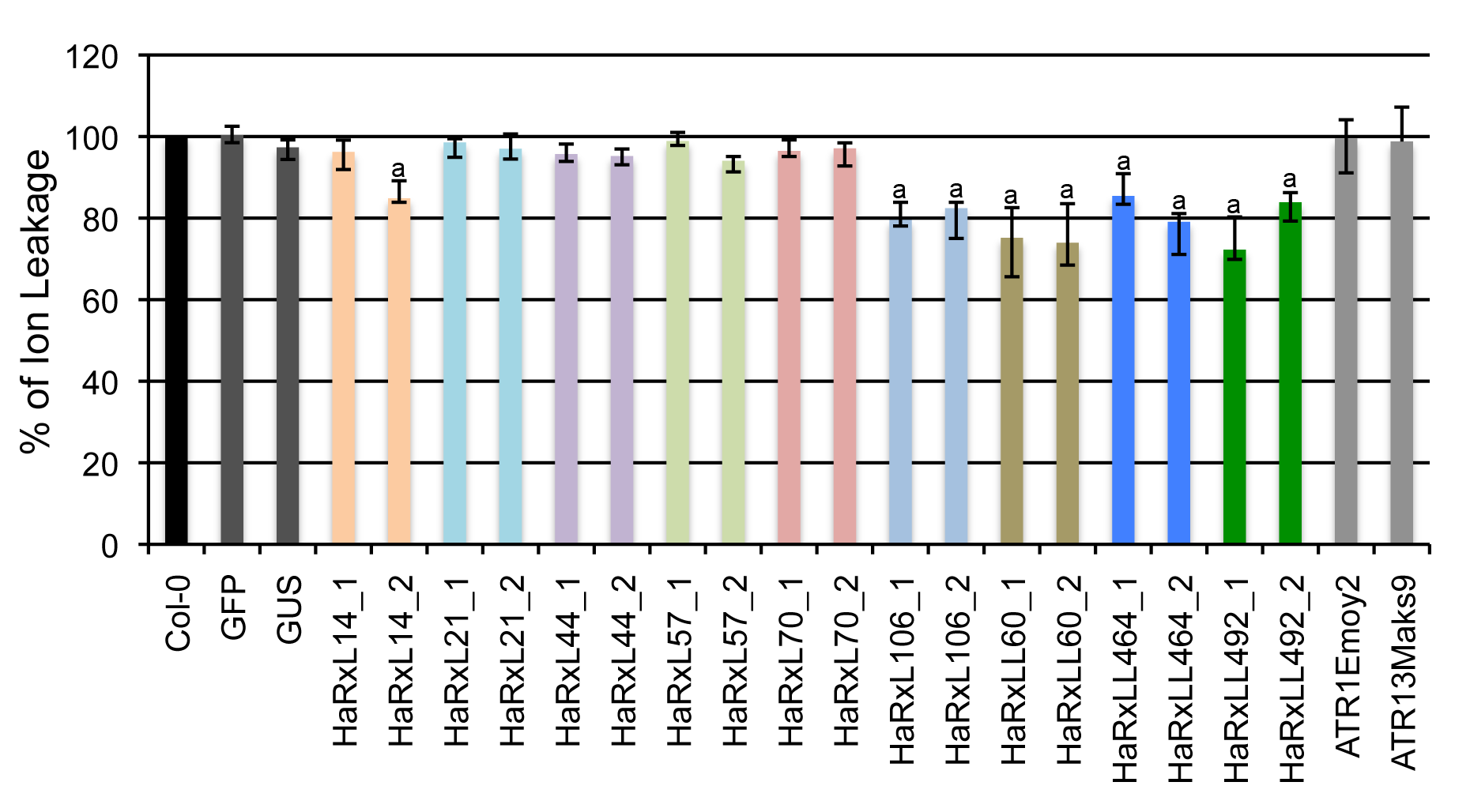

Supplement: Figure S7 — Constitutive expression of four different HaRxLs caused a mild reduction on the levels of ion leakage triggered by AvrRPM1 recognition. Five-week-old plants were hand infiltrated with Pf0-1 delivering P. maculicola AvrRPM1 at OD600 = 0.1. Twenty-four leaf discs were sampled from four infiltrated transgenic plants per line per HaRxL in each of five different experiments. At least six technical replicates were done per line. Conductivity was measured in the water were discs were floating, as an indication of ion leakage into the media. Measurements were taken every hour until the peak of ion leakage was detected in the wild type and control lines (around 14 hours post-infiltration). Bars correspond to the average ±2× SE of the maximum level of ion leakage observed for each line expressed as a percentage of the value displayed by Col-0 wild type (100%). Values take into account averaged results of 5 different experiments. (a) p value<0.05 of two tailed Z-test. (TIF) [file ppat.1002348.s007.tif]

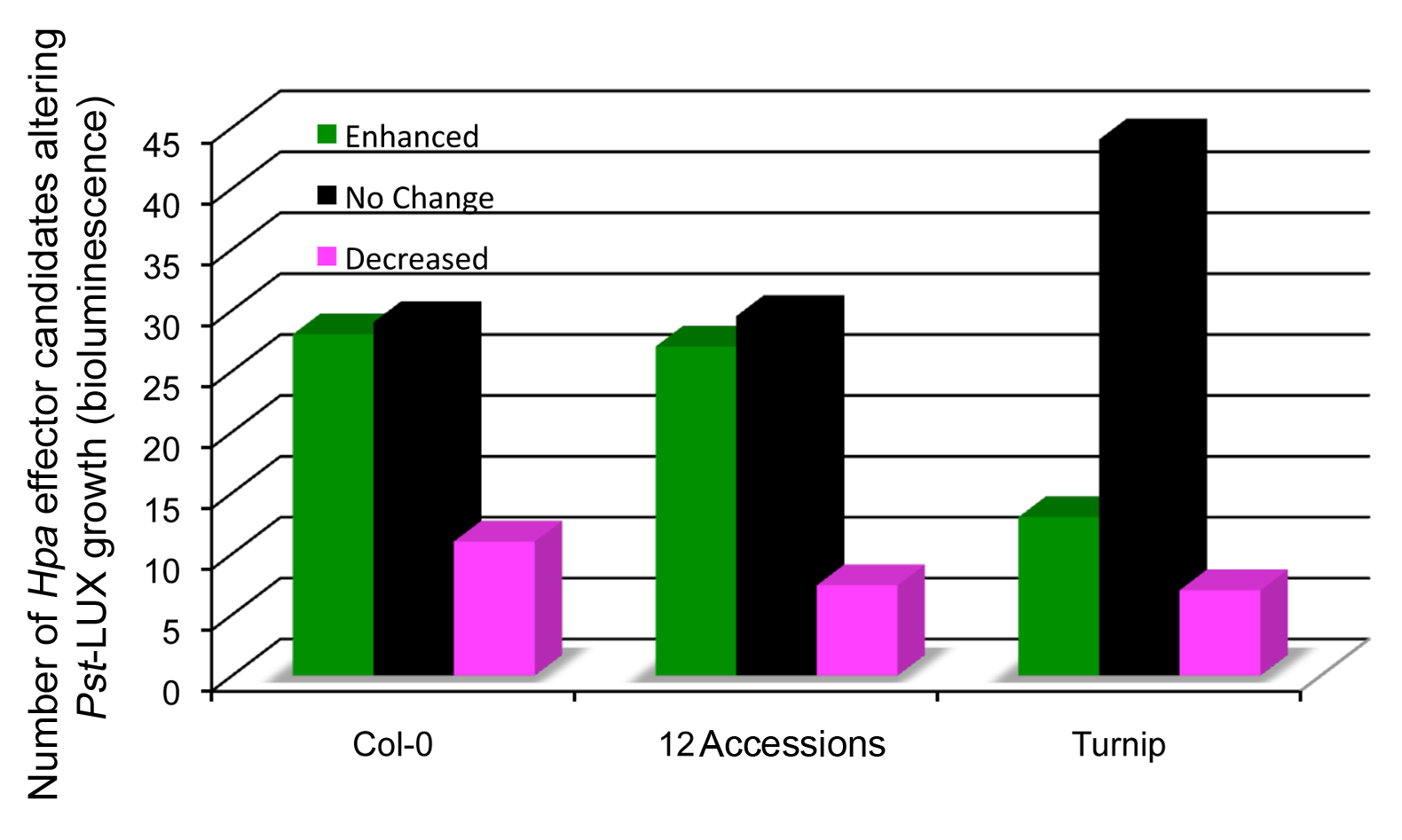

Supplement: Figure S8 — Fewer HaRxLs can alter the growth of Pst -LUX in the Hpa -non-host Brassica rapa compared to Arabidopsis . Columns show the distribution of the number of candidate effectors that enhance or decrease Pst-LUX virulence in the non-host B. rapa compared to one accession (Col-0) and the average results of the screening in the set of twelve accessions of the host Arabidopsis. (TIF) [file ppat.1002348.s008.tif]
