# Supplementary material for: Multiple Candidate Effectors from the Oomycete Pathogen Hyaloperonospora arabidopsidis Suppress Host Plant Immunity
Source: PLoS Pathog. 2011 Nov 3;7(11):e1002348. doi: 10.1371/journal.ppat.1002348 (PMC3207932; doi:10.1371/journal.ppat.1002348)
Supplement: Table S3 — Comparison of photon counts versus colony forming units for Pst -LUX. Results obtained delivering 13 Hpa candidate effectors via EDV by Pst-LUX in different Arabidopsis accessions. Numbers in the body of the table correspond to the ratio between Pst-LUX delivering the indicated effector and the control Pst-LUX clone delivering YFP. For the spray inoculation, the quantization of the bacterial bioluminescence (Photon Counts) and colony forming units (CFUs) is displayed for two to 3 replicate experiments. Experiments displaying the results obtained when the bacteria are introduced in the plant by syringe infiltration are shown for comparison. For the spray inoculation experiments, bacterial growth was scored at 3 dpi using five plants to record Pst-LUX bioluminescence. The same plants were ground to determine bacterial growth by plating in selective media and CFUs. Experiments in parallel were done inoculating the bacteria at OD600: 0.001 by syringe infiltration. Numbers highlighted in bold indicate T-test p value<0.05. (*) Result differs from the one obtained with the EDV screen (see Table S2). (a) Values of photon counts correspond to the ratio of counts per second (CPS)/fresh weight (FW) in grams of Pst-LUX clones delivering via EDV the stated Hpa candidate effectors versus CPS/FW of the YFP or AvrRPS4AAAA control. (b) Values of CFUs correspond to the ratio colony forming units (CFUs)/FW of Pst-LUX clones delivering via EDV the stated Hpa candidate effectors versus CFUs/FW of the YFP control or AvrRPS4AAAA control. (c) The concordance between the Photon counts and CFUs ratios is indicated. (DOC) [file ppat.1002348.s011.doc]

Table S3. Comparison of photon counts *versus* colony forming units for *Pst*-LUX

| *Arabidopsis* | *Hpa* | Spray Inoculation | | | | | | Infiltration | | Concordance ^(c)^ | |
| --- | --- | --- | --- | --- | --- | --- | --- | --- | --- | --- | --- |
| Accession | Effector | Photon Counts ^(a)^ | | | CFUs ^(b)^ | | | CFUs ^(b)^ | | Spray | Infiltration |
|  |  | E1 | E2 | E3 | E1 | E2 | E3 | E1 | E2 |  |  |
| Col-0 | HaRxL21 | **1.56** | **1.67** | **1.45** | **4.66** | **6.23** | **2.15** | **2.55** | **2.58** | Yes | Yes |
| Col-0 | HaRxL106 | **0.56** | **0.48** | **0.78** | **0.14** | 0.22 | 0.66 | **0.13** | **0.15** | Yes | Yes |
| Col-0 | HaRxL57 | **1.40** | **2.80** | **2.60** | **4.43** | **10.53** | **2.17** | **3.78** | **2.95** | Yes | Yes |
| Col-0 | HaRxLL492 | 1.35 | **1.66** | **1.51** | 1.11 | **3.85** | **2.70** | **2.90** | **2.65** | Yes | Yes |
| Col-0 | HaRxL70 | 0.98 | 1.21 | 1.18 | 0.67 | 0.78 | 0.88 | 0.92 | 0.81 | Yes | Yes |
| Col-0 | HaRxLL464 | **2.03** | 1.26 | **2.18** | **7.39** | 1.79 | **4.67** | **6.33** | **4.13** | Yes | Yes |
| Col-0 | HaRxLL60 | 1.27 | **1.89** | **1.76** | **2.41** | **7.24** | **7.73** | **3.73** | **4.71** | Yes | Yes |
| Col-0 | HaRxL44 | 1.45 | **2.15** | **2.57** | **3.99** | **6.07** | **13.30** | **6.51** | **7.19** | Yes | Yes |
| Col-0 | HaRxL14 | **2.04** | **2.08** | **1.97** | **3.61** | **5.15** | **4.25** | **3.19** | **4.80** | Yes | Yes |
| Col-0 | ATR13^Emco5^ | 1.12 | **2.35** | **2.16** | 1.47 | **3.64** | **3.93** | **2.05** | **2.71** | Yes | Yes |
| Col-0 | HaRxL79 | 1.12 | **0.64** | **0.81** | **0.15** | **0.40** | **0.16** | 0.93 | 1.15 | Yes | No |
| Col-0 | HaRxL62 | **2.19** | **2.11** | **1.72** | **4.56** | **5.46** | **4.54** | **4.19** | **9.17** | Yes | Yes |
| Ksk-1 | HaRxL57 | 0.72 | **0.56** | **0.44** | 1.02 | **0.61** | **0.72** | ND | ND | Yes | ND |
| Ksk-1 | HaRxLL60 | 1.37 | **2.59** | **1.44** | 0.91 | **4.12** | **4.97** | ND | ND | Yes | ND |
| Ksk-1 | HaRxL45 | 1.01 | 0.87 |  | 0.92 | 0.90 |  | ND | ND | Yes | ND |
| Ler-0 | HaRxL44 | 0.76 | **0.38** | 0.82 | 0.72 | 0.77 | **0.56** | ND | ND | Yes | ND |
| Ler-0 | HaRxL62 | **6.85** | **3.32** | 1.35 | **5.46** | **3.57** | 1.33 | **4.19** | **5.32** | Yes | Yes |
| Ler-0 | HaRxL45 | 1.01 | 0.98 | 0.93 | 1.05 | 1.65 | 1.12 | ND | ND | Yes | ND |
| Nd-0 | HaRxL106 | **1.82** | **2.25** | **1.56** | **3.97** | **9.94** | **3.25** | ND | ND | Yes | ND |
| Nd-0 | HaRxL14 | **3.91** | **1.97** |  | **13.55** | **6.25** |  | ND | ND | Yes | ND |
| Nd-0 | HaRxL62 | **2.24** | **2.64** | **3.31** | **4.16** | **3.89** | **3.93** | ND | ND | Yes | ND |
| Nd-0 | ATR13^Emco5^ | **0.34** | **0.36** |  | **0.13** | **0.20** |  | **0.11** | **0.13** | Yes | Yes |
| Oy-0 | HaRxL79 | **0.61** | 0.88 | **0.69** | **0.23** | **0.01** | **0.51** | 1.14 | 0.98 | Yes | No |
| Oy-0 | HaRxL21 | 1.44 | **1.98** | **2.31** | 1.73 | **3.74** | **10.21** | ND | ND | Yes | ND |
| Oy-0 | HaRxLL492 | 0.98 | 1.23 | 1.17 | 0.95 | 1.34 | 0.69 | ND | ND | Yes | ND |
| Oy-0 | HaRxL14 | **2.45** | **2.13** |  | **4.88** | **8.28** |  | ND | ND | Yes | ND |
| Ts-1 | HaRxL21 | **1.82** | **1.63** |  | **2.65** | **3.92** |  | ND | ND | Yes (*) | ND |
| Ts-1 | HaRxLL464 | 1.12 | 1.14 | 1.32 | 1.23 | 2.21 | 1.87 | ND | ND | Yes | ND |
| Ts-1 | HaRxLL60 | 1.1.7 | 0.92 | 0.98 | 0.86 | 0.96 | 1.13 | ND | ND | Yes | ND |
| Ts-1 | HaRxL44 | **0.67** | 0.82 | **0.45** | 1.25 | 1.39 | 0.82 | ND | ND | No | ND |
| Ws-0 | HaRxL21 | 1.94 | **0.47** | 0.98 | 0.73 | **0.42** | **0.59** | ND | ND | No | ND |
| Ws-0 | HaRxLL464 | 0.96 | 1.12 | 0.97 | 0.94 | 1.56 | 1.66 | ND | ND | Yes | ND |
| Ws-0 | HaRxLL60 | 1.15 | 1.30 | 0.89 | 0.76 | 1.23 | 1.15 | ND | ND | Yes | ND |
| Ws-0 | HaRxL106 | **2.17** | **2.59** |  | **3.68** | **4.12** |  | ND | ND | Yes | ND |

Results obtained delivering 13 *Hpa* candidate effectors via EDV by *Pst*-LUX in different *Arabidopsis* accessions. Numbers in the body of the table correspond to the ratio between *Pst*-LUX delivering the indicated effector and the control *Pst*-LUX clone delivering YFP. For the spray inoculation, the quantization of the bacterial bioluminescence (Photon Counts) and colony forming units (CFUs) is displayed for two to 3 replicate experiments. Experiments displaying the results obtained when the bacteria are introduced in the plant by syringe infiltration are shown for comparison. For the spray inoculation experiments, bacterial growth was scored at 3dpi using five plants to record *Pst*-LUX bioluminescence. The same plants were ground to determine bacterial growth by plating in selective media and CFUs. Experiments in parallel were done inoculating the bacteria at OD_600_: 0.001 by syringe infiltration. Numbers highlighted in bold indicate T-test p value <0.05. (*) Result differs from the one obtained with the EDV screen (see Table S2).

(a) Values of photon counts correspond to the ratio of counts per second (CPS)/ fresh weight (FW) in grams of *Pst*-LUX clones delivering via EDV the stated *Hpa* candidate effectors versus CPS/FW of the YFP or AvrRPS4^AAAA^ control.

(b) Values of CFUs correspond to the ratio colony forming units (CFUs)/FW of *Pst*-LUX clones delivering via EDV the stated *Hpa* candidate effectors versus CFUs/FW of the YFP control or AvrRPS4^AAAA^ control.

(c) The concordance between the Photon counts and CFUs ratios is indicated.
