# Supplementary material for: Multiple Candidate Effectors from the Oomycete Pathogen Hyaloperonospora arabidopsidis Suppress Host Plant Immunity
Source: PLoS Pathog. 2011 Nov 3;7(11):e1002348. doi: 10.1371/journal.ppat.1002348 (PMC3207932; doi:10.1371/journal.ppat.1002348)
Supplement: Table S4 — Putatively recognized Hpa candidate effectors and HR-like cell death. The table displays the number of leaves showing HR-like micro-lesions when Trypan blue-stained 24 h post-infiltration with Pst-ΔCEL (OD600 = 0.01) or Pf0-1 (OD600 = 0.1) delivering the indicated Hpa RXLR effector candidates in the corresponding Arabidopsis accessions. Numbers in parenthesis correspond to Pf0-1 data. Twenty-four leaves were infiltrated per candidate per bacterium. This experiment was repeated twice for each vehicle bacterium, with similar results. Values significantly different from negative controls (NC1 or NC2) across replicate experiments are indicated in bold (p value T-test<0.05). (DOC) [file ppat.1002348.s012.doc]

Table S4. Putatively recognized *Hpa* candidate effectors and HR-like cell death.

| **Effector** | **Bay-0** | **Br-0** | **Col-0** | **Ksk-1** | **Ler-0** | **Nd-0** | **Oy-0** | **Sha** | **Ts-1** | **Tsu-1** | **Wei-0** | **Ws-0** |
| --- | --- | --- | --- | --- | --- | --- | --- | --- | --- | --- | --- | --- |
| ATR13^Emco5^ |  |  |  |  |  | **18 (16)** |  |  |  |  |  |  |
| ATR13^Emoy2^ |  |  |  |  |  |  | 2 (3) |  |  |  |  |  |
| HaRxLL495 |  |  |  |  |  |  |  | 4 (5) |  |  | 3 (2) |  |
| **HaRxL4** |  |  | **16 (15)** |  |  |  |  |  |  |  |  |  |
| HaRxL106 |  |  | **14** (2) | **20** (3) |  |  |  |  |  |  |  |  |
| HaRxL67 |  |  | 4 (1) |  |  |  | 4 (3) | 3 (2) |  |  | 4 (2) |  |
| **HaRxL70** | **11 (13)** |  |  |  |  |  |  |  |  |  |  |  |
| HaRxL45 | 3 (2) |  | 8 (10) |  |  |  |  | 2 (2) |  |  |  |  |
| HaRxL44 |  |  |  |  | 4 (3) |  |  |  | 5 (3) |  |  |  |
| HaRxL108 |  |  | 3 (4) | 3 (2) |  |  |  |  |  |  |  |  |
| HaRxL77 | 3 (2) |  | 2 (3) |  |  |  |  | 4 (3) | 4 (5) | 3 (2) |  |  |
| HaRxL13 |  |  |  |  | 4 (5) |  |  |  |  |  |  |  |
| HaRxL79 | 3 (2) | 2 (1) | 3 (2) |  | 3 (5) |  | 4 (3) | 5 (3) |  | 4 (4) |  |  |
| HaRxLL429 |  |  | 3 (4) |  |  |  | 4 (3) | 1 (4) |  |  |  |  |
| HaRxLL445 |  |  |  |  | 2 (3) |  |  |  |  |  |  |  |
| HaRxLL483 | 3 (4) |  |  |  | 3 (4) |  |  |  | 5 (3) |  | 5 (3) |  |
| HaRxLL441 |  |  |  |  |  |  |  | 3 (4) |  | 2 (2) |  | 3 (4) |
| HaRxL89 | 3 (2) |  |  |  | 4 (2) |  |  |  |  |  |  |  |
| HaRxL57 |  |  |  | 5 (4) |  |  |  |  |  |  |  |  |
| HaRxLL437 |  |  |  |  |  |  | 4 (3) |  | 4 (2) | 3 (3) |  |  |
| **HaRxL18** | **10 (12)** |  |  |  |  |  | 2 (4) |  | 3 (5) |  |  | **12 (10)** |
| HaRxL11 |  |  |  | 3 (4) |  |  |  |  | 5 (5) |  |  |  |
| **HaRxL80** | 2 (3) |  | 1 (2) | 2 (3) |  |  | **7 (9)** | **7 (11)** |  | 2 (3) |  |  |
| HaRxL147 |  |  |  |  |  |  | 2 (3) |  |  |  |  |  |
| HaRxL36 |  |  |  |  | 5 (3) |  | 4 (2) | 2 (2) |  |  | 4 (5) |  |
| HaRxLL470 |  |  |  |  | 2 (3) |  |  | 2 (3) | 1 (2) | 3 (4) | NT | NT |
| HaRxLL90 | 4 (5) |  |  |  |  |  | 4 (3) | 1 (3) |  |  |  |  |
| HaRxL74 |  |  | 2 (3) |  | 2 (3) |  | 1 (4) | 4 (3) | NT | 2 (2) | 1 (3) | 1 (3) |
| NC1 | 3 (2) |  | 3 (2) |  | 3 (2) |  | 2 (2) |  | 3 (1) |  | 3 (2) |  |
| NC2 |  | 3 (1) |  | 2 (3) |  | 2 (3) |  | 4 (4) |  | 5 (2) |  | 2 (2) |

The table displays the number of leaves showing HR-like micro-lesions when Trypan blue-stained 24 h post-infiltration with *Pst*-ΔCEL (OD_600_ = 0.01) or Pf0-1 (OD_600_ = 0.1) delivering the indicated *Hpa* RXLR effector candidates in the corresponding *Arabidopsis* accessions. Numbers in parenthesis correspond to Pf0-1 data. Twenty-four leaves were infiltrated per candidate per bacterium. This experiment was repeated twice for each vehicle bacterium, with similar results. Values significantly different from negative controls (NC1 or NC2) across replicate experiments are indicated in bold (p value T-test <0.05)
